# Novel cellular functions of Cys_2_-His_2_ zinc finger proteins in anthracnose development and dissemination on pepper fruits by *Colletotrichum scovillei*

**DOI:** 10.1128/mbio.00667-24

**Published:** 2024-09-09

**Authors:** Teng Fu, Yong-Won Song, Guoyang Gao, Kyoung Su Kim

**Affiliations:** 1 Division of Bio-Resource Sciences, Interdisciplinary Program in Smart Agriculture, and Bioherb Research Institute, Kangwon National University, Chuncheon, South Korea; Yonsei University, Seoul, South Korea

**Keywords:** *Colletotrichum scovillei*, pepper anthracnose, Cys2-His2 zinc finger protein, appressorium development, host defense

## Abstract

*Colletotrichum* species are notorious for causing anthracnose on many fruits, leading to significant economic losses worldwide. As a model, we functionally characterized cys2-his2 (C_2_H_2_) zinc finger proteins (CsCZFs) in *Colletotrichum scovillei*, a major causal agent of pepper fruit anthracnose in many countries. In all, 62 CsCZFs were identified by *in silico* genomic analysis. Twelve were selected based on their expression profiles to generate targeted deletion mutants for functional investigation. *ΔCsczf1* markedly reduced conidiation and constitutive expression of *CsCZF1* partially recovered conidiation in an asexual reproduction-defective mutant, *ΔCshox2*. Deletion of *CsCZF12*, orthologous to the calcineurin-responsive transcription factor *Crz1*, impaired autophagy in *C. scovillei. ΔCsczf9* was defective in surface recognition, appressorium formation, and suppression of host defenses. *CsCZF9* was identified as an essential and novel regulator under the control of the mitogen-activated protein kinase (CsPMK1) in an early step of appressorium development in *C. scovillei*. This study provides novel insights into *CsCZF*-mediated regulation of differentiation and pathogenicity in *C. scovillei*, contributing to understanding the regulatory mechanisms governing fruit anthracnose epidemics.

**IMPORTANCE:**

The phytopathogenic fungus *Colletotrichum scovillei* is known to cause serious anthracnose on chili pepper. However, the molecular mechanism underlying anthracnose caused by this fungus remains largely unknown. Here, we systematically analyzed the functional roles of cys2-his2 zinc finger proteins (CsCZFs) in the dissemination and pathogenic development of this fungus. Our results showed that CsCZF1 plays an important role in conidiation and constitutive expression of CsCZF1 restored conidiation in an asexual reproduction-defective mutant, *ΔCshox2*. The CsCZF9, a novel target of the mitogen-activated protein kinase (CsPMK1), is essential for surface recognition to allow appressorium formation and suppression of host defenses in *C. scovillei*. The CsCZF12, orthologous to the calcineurin-responsive transcription factor Crz1, is involved in the autophagy of *C. scovillei*. Our findings reveal a comprehensive mechanism underlying CsCZF-mediated regulation of differentiation and pathogenicity of *C. scovillei*, which contributes to the understanding of fruit anthracnose epidemics and the development of novel strategies for disease management.

## INTRODUCTION

Anthracnose, caused by species belonging to the ascomycete genus *Colletotrichum*, is a cosmopolitan disease of many plants, which poses a serious threat to the agricultural productivity of many fruits worldwide (1, 2). Practices for the management of anthracnose disease rely mainly on multiple applications of chemical fungicides during the growing season (3). Several different species of *Colletotrichum* cause anthracnose disease, which is a substantial obstacle to the development of resistant cultivars and disease management (1, 4–6). Therefore, understanding the molecular biology underlying polycyclic dissemination and plant infection of *Colletotrichum* species is a prerequisite for novel strategies for anthracnose control. *Colletotrichum scovillei* is a major pathogen that infects economically important fruits and is a dominant species on pepper fruits (7–9). The infected fruits typically exhibit sunken necrotic spots of anthracnose within a few days, leading to considerable damage to pepper production (9).

The pathogen produces massive conidia in a polycyclic infection, which develops appressoria on the tips of germ tubes upon host recognition (9). Unlike foliar fungal pathogens, which show direct penetration into host epidermal cells, *C. scovillei* forms compact, bulbous hyphae inside the thick cuticle layer of pepper fruits before invading host epidermal cells, which is visualized and designated as a dendroid structure (10). This may represent a special infection and survival strategy of *C. scovillei* beneath the subcuticular layer that is distinct from foliar fungal pathogens (10, 11). Despite the severity of fruit productivity problems caused by *Colletotrichum* species, our understanding of the nature of anthracnose on fruits is limited. Cellular events involved in conidiation and appressorium formation are key steps affecting propagule dissemination and disease development. Previously, the deletion of *CsHOX2*, encoding a homeobox transcription factor (TF), was shown to cause a complete conidiation defect in *C. scovillei*. Mitogen-activated protein kinase 1 (Pmk1) is a key regulator of appressorium development (12–18). The deletion of *CsPMK1* causes defects in both appressorium formation and surface recognition in *C. scovillei* (15), and Pmk1 orchestrates several cellular processes for appressorium function by regulating other effectors, such as TFs and proteins (19, 20).

In this study, we attempted to find other TFs associated with Pmk1 during the development of *C. scovillei*. Members of the cys2-his2 (C_2_H_2_) zinc finger protein family, characterized by the zinc finger domain composed of 20–30 amino acid residues, control the expression of target genes by binding to specific DNA sequences (21, 22). In fungal plant pathogens, a number of C_2_H_2_ zinc finger proteins (CZFs) are critical regulators, as they play important roles in fungal growth, conidiation, appressorium formation, survival, stress adaption, and plant infection (23–28). Thus, understanding their functional basis is crucial to unravel the comprehensive physiological processes that regulate developmental events. However, their functions have not been investigated in *C. scovillei*. In this study, we searched for genes encoding CsCZFs in the genome of *C. scovillei* and evaluated their expression during fungal development and infection. We identified 62 CsCZFs and classified them into three groups based on their expression. We further selected 12 of them (*CsCZF1–12*) to characterize their functions using targeted deletion mutants. Importantly, *ΔCsczf9* was defective in surface recognition, appressorium formation, and suppression of host defenses, similar to *ΔCspmk1* (15). CsCZF9 was identified as a novel TF target of CsPMK1. In addition, *CsCZF1* was found to be important for conidiation, and its constitutive expression partially restored conidiation of *ΔCshox2*, a previously reported deletion mutant defective in asexual reproduction (9). A novel function of CsCZF12/CRZ1, orthologous to the calcineurin-responsive transcription factor Crz1, was found to regulate autophagy. Our results provide novel insights into the regulatory roles of CsCZFs in terms of dissemination, survival, and anthracnose development of *C. scovillei*.

## RESULTS

### Isolation and expression of CsCZFs

In all, 105 CsCZFs were identified by searching the whole-genome sequence of *C. scovillei* strain KC05 (Fig. S1). Among them, 62 exclusively possessed the C_2_H_2_-type zinc finger domain (InterPro term: IPR013087) (Fig. S1). With the exception of CAP_004582.1, the other 61 CsCZFs have orthologs present in 14 selected in-group species of the *C. acutatum* species complex (CASC) (Table S1). The genome of *C. scovillei* was found to contain more CsCZFs than *Saccharomyces cerevisiae* and several other plant pathogenic fungi (Fig. S1).

To predict the functions of CsCZFs in *C. scovillei*, quantitative reverse transcriptase polymerase chain reaction (qRT-PCR) was performed to evaluate their expressions in conidia (CO), appressoria (AP), and infectious hyphae at 24 h (IF-24) and 72 h (IF-72), compared to mycelia (MY). The gene expression profiles revealed that 62 *CsCZFs* were grouped into three clusters (CluI: 22 genes, CluII: 19 genes, and CluIII: 21 genes) (Fig. S2). The *CsCZFs* in CluI and CluIII showed differential expressions and were selected for further study. In CluI, six genes exhibited extremely high expressions (>20-fold) in CO, compared to MY, AP, IF-24, and IF-72. Among these, four genes (CAP_005682.1, CAP_009446.1, CAP_003899.1, and CAP_006607.1) with orthologs in both in-group and out-group species of the CASC were selected to study their roles in conidiation of *C. scovillei*. In CluIII, 12 genes exhibited high expression levels (>4-fold) in AP, IF-24, or IF-72, compared to MY. Two of these genes (CAP_003653.1 and CAP_006533.1) with high expressions in AP were chosen to investigate their involvement in the appressorium development of *C. scovillei*. For the remaining 10 genes highly expressed in IF-24 and/or IF-24, six genes (CAP_011132.1, CAP_000892.1, CAP_005482.1, CAP_000583.1, CAP_012989.1, CAP_011662.1), whose orthologs were previously reported to be upregulated in plant infection of at least a *Colletotrichum* species (Table S2), were selected to explore their functions in infectious hyphae growth of *C. scovillei*. In total, 12 *CsCZFs* (*CsCZF1* to *CsCZF12/CRZ1*) were selected for functional characterization.

### Generation of CsCZF deletion mutants

The 12 *CsCZF* genes were deleted *via* homology-dependent replacement (Fig. S3). The deletion mutants and complemented transformants were confirmed by Southern blotting and RT-PCR, respectively (Fig. S3). All deletion mutants other than *ΔCsczf6* were defective in growth, development, and pathogenicity, compared to the wild-type and corresponding complemented strains (Table 1). Briefly, *ΔCsczf3*, *ΔCsczf5*, *ΔCsczf8*, *ΔCsczf10*, and *ΔCsczf12/crz1* were impaired in mycelial growth; *ΔCsczf1*, *ΔCsczf2*, *ΔCsczf3*, *ΔCsczf4*, *ΔCsczf8*, *ΔCsczf9*, *ΔCsczf10*, *ΔCsczf11*, and *ΔCsczf12/crz1* showed reduced conidiation; *ΔCsczf3*, *ΔCsczf8*, *ΔCsczf10*, and *ΔCsczf12/crz1* produced conidia with abnormal morphology; *ΔCsczf9* and *ΔCsczf12/crz1* were defective in appressorium formation and pathogenicity.

**TABLE 1:** Summary of phenotypes of *CsCZF* gene deletion mutants^*a*^

Strains	Growth (mm)^*b*^	Conidiation (10^4^/mL)^*c*^	Conidium size^*d*^		Conidial germination (%)^*e*^	Appressorium formation (%)^*f*^
			Length (µm)	Width (µm)		
Wild type	39.2 ± 0.8^DEF^	77.5 ± 2.7^G^	11.0 ± 0.9^BC^	3.7 ± 0.5^B^	88.5 ± 6.4^B^	86.5 ± 4.7^C^
*ΔCsczf1*	38.2 ± 0.3^CDE^	0.8 ± 0.7^A^	10.7 ± 1.1^B^	3.7 ± 0.4^B^	91.7 ± 5.0^B^	85.5 ± 3.0^C^
*Csczf1c*	39.3 ± 1.3^DEF^	75.7 ± 3.9^G^	11.3 ± 1.8^C^	3.7 ± 0.5^B^	88.3 ± 3.0^B^	85.0 ± 3.6^C^
*ΔCsczf2*	37.5 ± 0.9^CD^	60.0 ± 7.7^F^	11.3 ± 1.2^C^	3.7 ± 0.3^B^	87.5 ± 6.2^B^	87.0 ± 2.4^C^
*Csczf2c*	37.5 ± 0.9^DEF^	76.3 ± 3.9^G^	10.9 ± 0.8^BC^	3.8 ± 0.2^B^	88.8 ± 3.4^B^	85.5 ± 2.4^C^
*ΔCsczf3*	36.3 ± 0.3^BC^	28.3 ± 4.1^C^	14.2 ± 2.0^D^	4.4 ± 0.4^C^	91.5 ± 5.1^B^	86.8 ± 3.3^C^
*Csczf3c*	39.3 ± 0.3^DEF^	75.7 ± 6.0^G^	10.9 ± 1.7^BC^	3.8 ± 0.6^B^	88.7 ± 3.1^B^	86.3 ± 2.1^C^
*ΔCsczf4*	38.0 ± 1.3^CDE^	45.8 ± 5.8^D^	11.3 ± 1.4^C^	3.7 ± 0.4^B^	88.8 ± 6.5^B^	86.5 ± 2.9^C^
*Csczf4c*	39.3 ± 0.8^DEF^	75.3 ± 4.6^G^	10.8 ± 1.0^B^	3.8 ± 0.5^B^	88.3 ± 2.9^B^	86.3 ± 2.6^C^
*ΔCsczf5*	36.3 ± 0.6^BC^	78.7 ± 8.3^G^	11.2 ± 1.7^C^	3.7 ± 0.4^B^	91.7 ± 2.0^B^	85.2 ± 2.6^C^
*Csczf5c*	39.3 ± 1.2^DEF^	76.3 ± 7.9^G^	11.1 ± 1.4^C^	3.8 ± 0.3^B^	88.0 ± 2.6^B^	87.5 ± 2.9^C^
*ΔCsczf6*	39.5 ± 1.5^EF^	80.0 ± 8.4^G^	10.6 ± 1.6^B^	3.7 ± 0.8^B^	88.3 ± 4.5^B^	86.7 ± 4.7^C^
*Csczf6c*	39.5 ± 0.9^EF^	74.8 ± 4.7^G^	11.0 ± 1.1^BC^	3.8 ± 0.6^B^	88.5 ± 2.7^B^	87.3 ± 2.9^C^
*ΔCsczf7*	39.2 ± 0.6^DEF^	79.2 ± 5.8^G^	11.2 ± 1.5^C^	3.7 ± 0.4^B^	89.5 ± 4.7^B^	85.7 ± 4.2^C^
*Csczf7c*	39.5 ± 0.9^EF^	73.8 ± 2.6^G^	11.1 ± 1.6^BC^	3.8 ± 0.4^B^	88.7 ± 2.3^B^	85.7 ± 3.2^C^
*ΔCsczf8*	35.3 ± 1.3^B^	12.3 ± 2.7^B^	14.2 ± 2.5^D^	4.3 ± 0.4^C^	89.3 ± 5.7^B^	84.8 ± 3.8^C^
*Csczf8c*	39.3 ± 0.6^DEF^	74.3 ± 2.5^G^	11.2 ± 1.2^C^	3.7 ± 0.3^B^	89.0 ± 3.8^B^	86.2 ± 3.2^C^
*ΔCsczf9*	39.3 ± 1.5^DEF^	3.3 ± 1.2^A^	10.9 ± 1.2^BC^	3.7 ± 0.4^B^	88.5 ± 4.7^B^	0^A^
*Csczf9c*	39.3 ± 1.3^DEF^	76.2 ± 4.3^G^	10.9 ± 0.9^BC^	3.8 ± 0.2^B^	88.3 ± 1.6^B^	86.2 ± 2.6^C^
*ΔCsczf10*	36.8 ± 1.2^BC^	14.0 ± 2.1^B^	14.8 ± 1.0^D^	4.4 ± 0.6^C^	88.5 ± 4.9^B^	86.5 ± 3.5^C^
*Csczf10c*	39.2 ± 1.0^DEF^	75.2 ± 4.4^G^	11.0 ± 1.3^BC^	3.8 ± 0.3^B^	88.3 ± 2.7^B^	86.7 ± 3.9^C^
*ΔCsczf11*	40.0 ± 1.8^EF^	51.7 ± 6.1^E^	11.1 ± 1.6^BC^	3.8 ± 0.4^B^	87.3 ± 7.6^B^	82.5 ± 5.2^C^
*Csczf11c*	39.2 ± 0.8^DEF^	75.7 ± 3.6^G^	11.5 ± 1.2^C^	3.8 ± 0.5^B^	89.3 ± 4.2^B^	84.3 ± 3.8^C^
*ΔCsczf12/crz1*	32.4 ± 1.5^A^	28.7 ± 3.1^C^	G_1_: 10.9 ±1.5^BC^; G_2_: 7.4 ±1.2^A^	G_1_: 3.8 ± 0.5^B^; G_2_: 3.4 ± 0.3^A^	44.8 ± 4.0^A^	22.2 ± 5.9^B^
*Csczf12c/crz1c*	39.2 ± 1.6^DEF^	75.5 ± 4.3^G^	10.8 ± 1.4^B^	3.7 ± 0.5^B^	88.7 ± 1.8^B^	86.3 ± 3.3^C^

^*a*^ Data are presented as means ± the standard deviations of three independent experiments, with three replicates per experiment. The same superscript capital letters in a column indicate no significant difference. The significant differences were estimated using Duncan’s test (*P* < 0.05).

^*b*^ Mycelial growth was measured at 5 days after culture on potato dextrose agar.

^*c*^ Conidiation was evaluated by counting the number of conidia harvested with 5 mL of sterilized distilled water from 6-day-old V8 juice agar medium.

^*d*^ Conidial size was determined by measuring the lengths and widths of at least 100 conidia. The conidia produced by *ΔCsczf12/crz1* were classified into two groups (G_1_ and G_2_).

^*e*^ Percentage of conidial germination was measured at 12 h post-inoculation on hydrophobic coverslips using conidia from 7-day-old oatmeal agar. In each replicate, at least 100 conidia were measured.

^*f*^ Percentage of appressorium formation was measured at 16 h post-inoculation on the hydrophobic coverslips using conidia from 7-day-old oatmeal agar. In each replicate, at least 100 conidia were measured.

### *CsCZF1* plays an important role in conidiation

**Fig 1:**
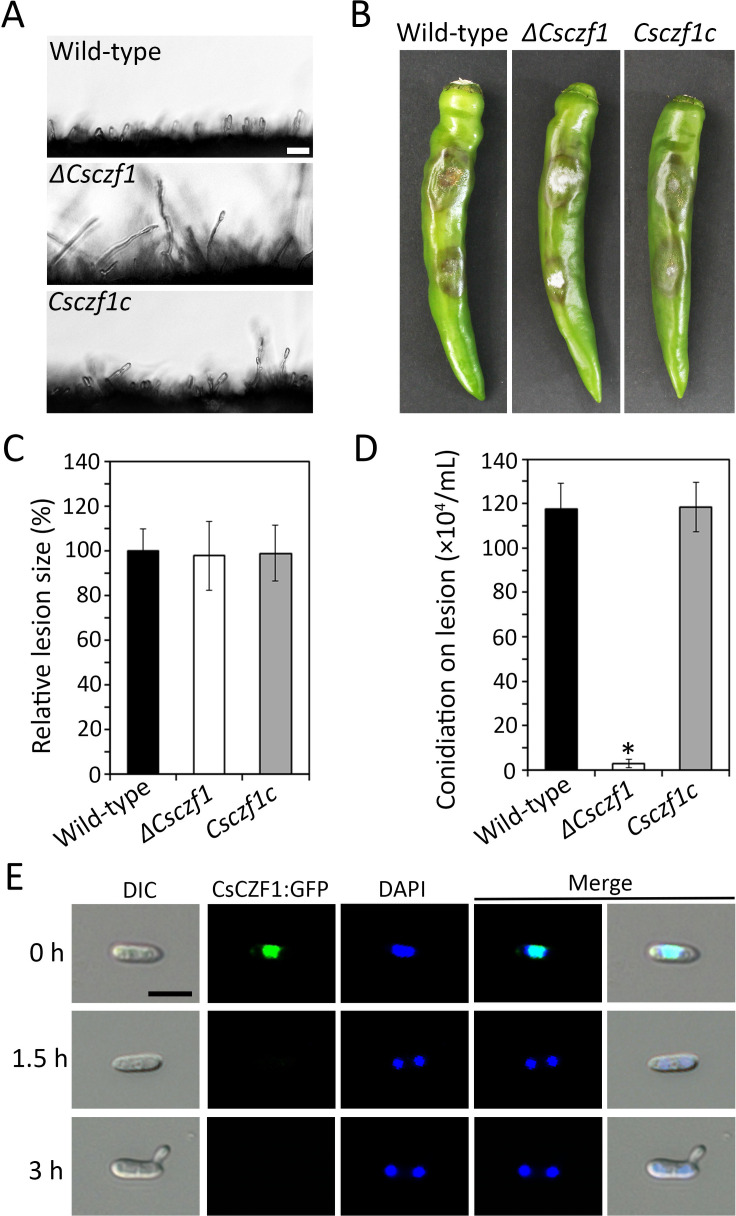
The *CsCZF1* is important for conidiation and localizes in nuclei. (**A**) Visualization of conidiation. Hyphal agar plugs from 3-day-old oatmeal agar were placed on a hydrophilic coverslip and incubated in a humid box at 25°C and light for 5 h. Scale bar = 20 µm. (**B and C**) Pathogenicity assay. (**B**) Photographs of anthracnose disease formation. Drops of conidial suspensions (8 × 10^4^ mL^−1^) were inoculated to intact pepper fruits and incubated in a humid box at 25°C. The photographs were taken after 11 days. (**C**) Quantitative measurement of anthracnose disease formation. The lesion size was measured using Image J and normalized to that caused by the wild type as a relative of 100%. (**D**) Conidiation on anthracnose lesions. Conidial suspensions (8 × 10^4^ mL^−1^) were inoculated to intact pepper fruits and incubated in a humid box at 25°C for 10 days. Reproduced conidia were rinsed with 3 mL of sterilized distilled water from the lesions. A significant difference was estimated by Duncan’s test (*P* < 0.05) and indicated by an asterisk (*). (**E**) Subcellular localization of CsCZF1. Conidial suspensions (5 × 10^4^ mL^−1^) from transformants expressing CsCZF1:GFP fusion protein were dropped to the hydrophobic coverslip and incubated in a humid box at 25°C. The nuclei were stained with DAPI. Scale bar = 10 µm.

**Fig 2:**
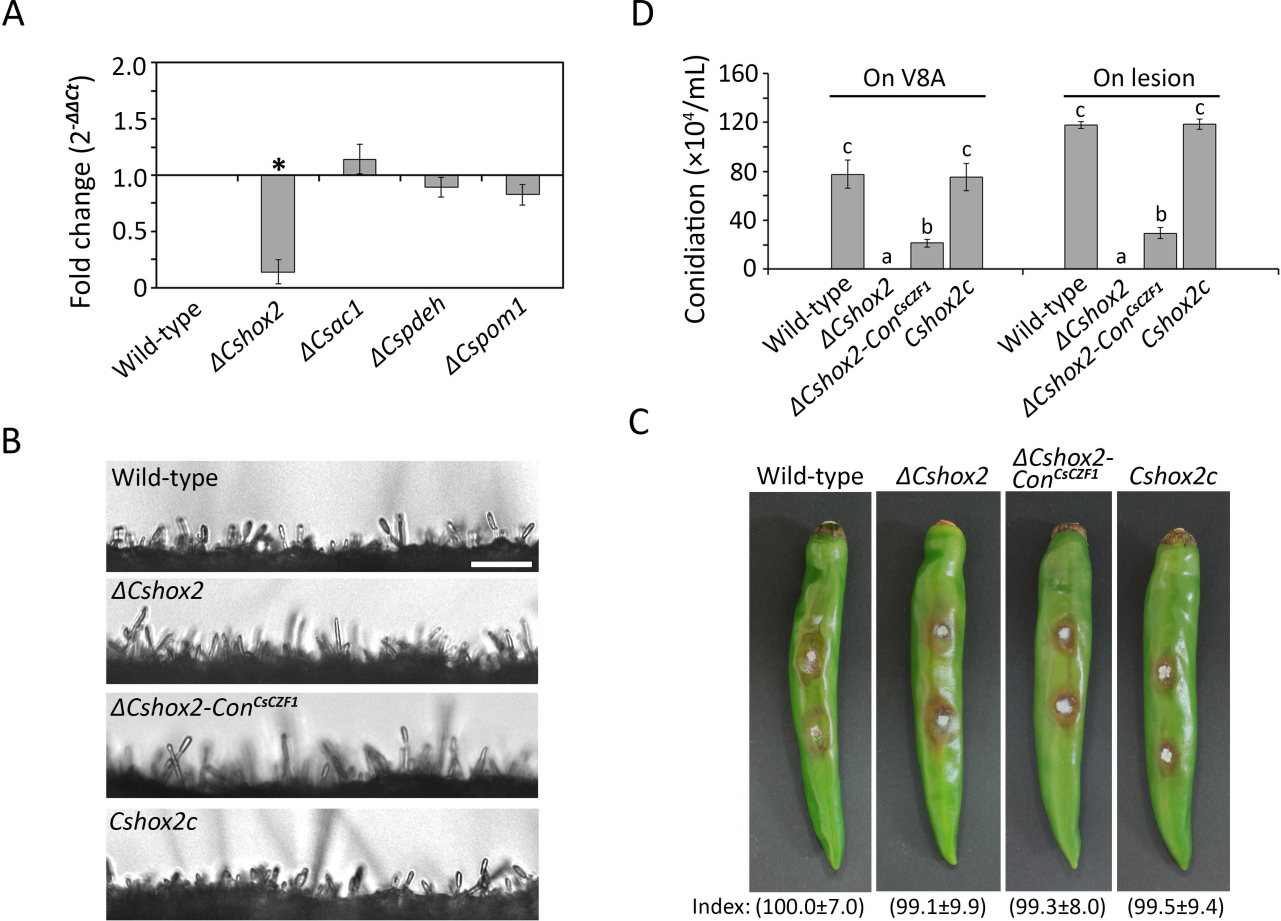
Constitutive expression of *CsCZF1* partially recovers conidiation of *ΔCshox2*. (**A**) Expression of *CsCZF1* in *C. scovillei* deletion mutants, which are defective in conidiation. Total RNA was extracted from fungal tissues during the conidiation stage. Expression of *CsCZF1* in deletion mutants was referenced to that in the wild-type strain. An asterisk (*) indicates the gene expression was differentially downregulated (Fold change <0.5). (**B**) Visualization of conidiation. Hyphal agar plugs from 3-day-old oatmeal agar were placed on the hydrophilic coverslip and incubated in a humid box at 25°C and light for 5 h. Scale bar = 40 µm. (**C**) Pathogenicity assay. Hyphal agar plugs (5 mm in diameter) from 3-day-old oatmeal agar were inoculated to wounded pepper fruits and incubated in a humid box at 25°C for 7 days. The lesion size was measured using Image J and normalized to that caused by the wild type as a relative of 100%. The values of relative lesion size are shown below in photographs. (**D**) Evaluation of conidiation. Wounded pepper fruits were inoculated with hyphal agar plugs (5 mm in diameter) from 3-day-old oatmeal agar and incubated in a humid box at 25°C for 7 days. Conidia were harvested with 5 and 3 mL of sterilized distilled water from 7-day-old V8 juice agar and lesions of wounded pepper fruits, respectively. A significant difference in a group was estimated by Duncan’s test (*P* < 0.05) and indicated by different lowercase letters.

*ΔCsczf1* showed normal mycelial growth, conidial germination, appressorium formation, and plant infection but markedly reduced conidiation compared to wild type and *Csczf1c* (Table 1; Fig. 1A through D). Microscopic observation showed that *ΔCsczf1* was able to develop conidiophores but was severely defective in the differentiation of conidia (Fig. 1A). These results suggest that CsCZF1 is a stage-specific regulator of conidiation in *C. scovillei*. To investigate the expression and localization of CsCZF1, a CsCZF1:green fluorescent protein (GFP) fusion protein was expressed in the wild-type strain. It was expressed in conidia but not germinating conidia and localized to the nucleus as evidenced by colocalization with the nuclear marker 4′,6-diamidino-2-phenylindole (DAPI) (Fig. 1E). Several genes, including *CsHOX2, CsAc1, CsPdeH, and CsPOM1*, have previously been shown to regulate conidiation in *C. scovillei* (9, 10, 29). Therefore, the expression of *CsCZF1* was evaluated in these deletion mutants by qRT-PCR. Its expression was highly downregulated only in *ΔCshox2* compared to the wild-type strain (Fig. 2A). Constitutive expression of *CsCZF1* in *ΔCshox2* partially recovered the defect in conidiation of *ΔCshox2* when cultured on both V8 juice agar medium and pepper fruits (Fig. 2B through D). These observations suggest that *CsCZF1* is regulated by *CsHOX2* to control conidiation in *C. scovillei*.

### *CsCZF3*, *CsCZF8*, and *CsCZF10* are related to conidium morphology and thermal stress adaption

*ΔCsczf3*, *ΔCsczf8*, and *ΔCsczf10* produced conidia with abnormal morphology but showed normal conidial germination, appressorium formation, and plant infection (Table 1; Fig. S4A). In *C. scovillei*, several deletion mutants producing morphologically abnormal conidia have been reported to be impaired in plant infection and stress adaption (9, 15, 29). Therefore, we examined whether *CsCZF3*, *CsCZF8*, and *CsCZF10* are involved in thermal stress tolerance. The *ΔCsczf3*, *ΔCsczf8*, and *ΔCsczf10* were found to be defective in conidium viability at 32°C compared to the wild-type and corresponding complemented strains (Fig. S4B). Expectedly, *ΔCsczf3*, *ΔCsczf8*, and *ΔCsczf10* caused anthracnose with significantly reduced severity to the wild-type and complemented strains on intact pepper fruits at 32°C (Fig. S4A). These results suggest that the *CsCZF3*, *CsCZF8*, and *CsCZF10* are involved in conidium morphology and tolerance to thermal stress.

### *CsCZF12/CRZ1* has multiple functions in fungal differentiation, stress adaption, and pathogenicity

**Fig 3:**
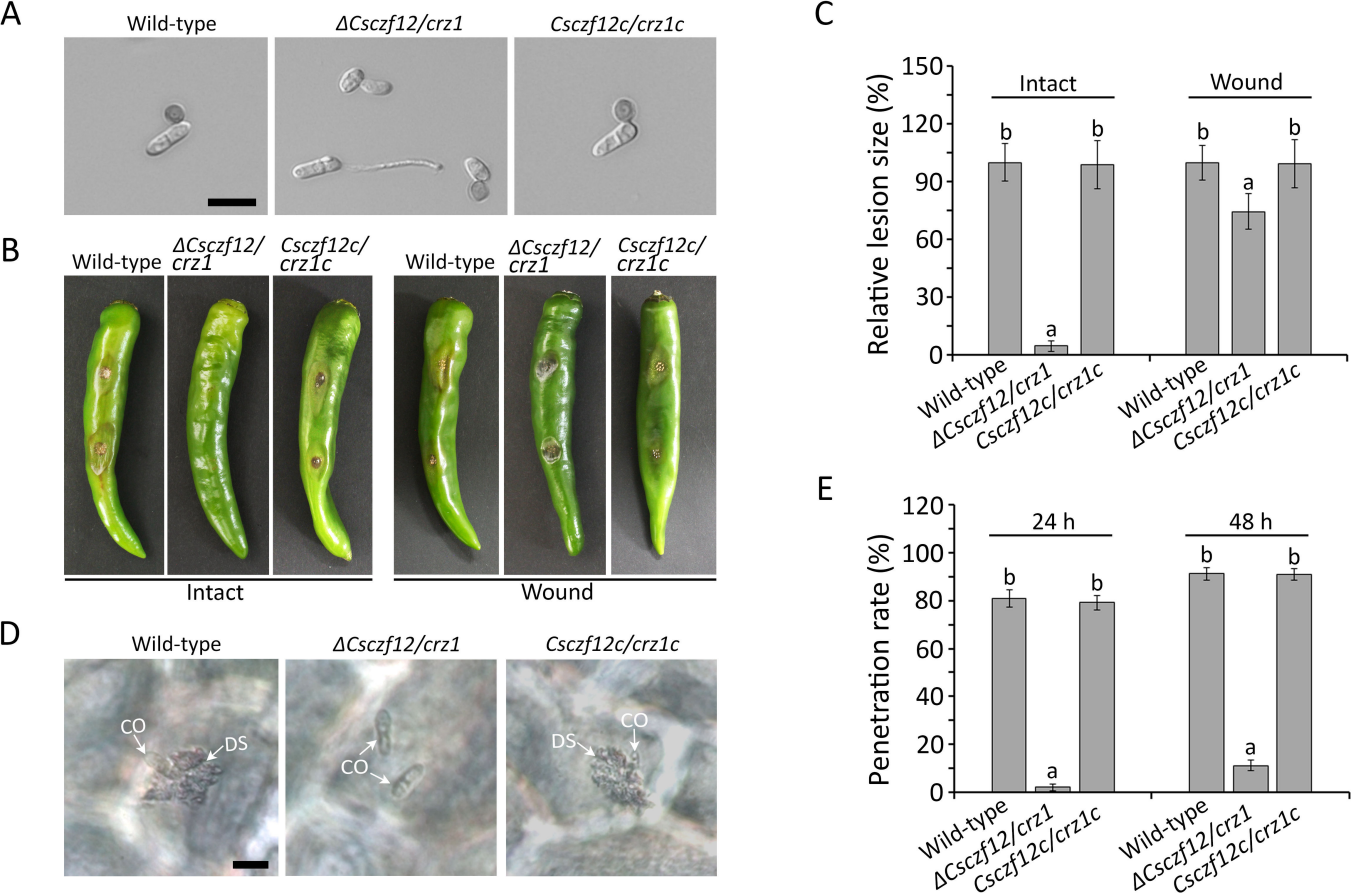
The *CsCZF12/CRZ1* plays important roles in fungal development and pathogenicity. (**A**) Appressorium formation. Drops of conidial suspensions (5 × 10^4^ mL^−1^) were placed on the hydrophobic coverslip and incubated in a humid box at 25°C for 16 h. Scale bar = 10 µm. (**B and C**) Pathogenicity assays. (**B**) Photographs of disease formation. Conidial suspensions (2.5 × 10^5^ mL^−1^) were inoculated to wounded and intact pepper fruits and incubated in a humid box for 6 and 8 days, respectively. (**C**) Quantitative measurement of disease formation. The lesion size was calculated using Image J and normalized to that caused by the wild-type strain as a relative of 100%. (**D and E**) Appressorium-mediated penetration. (**D**) Visualization of penetration. Conidial suspensions (5 × 10^5^ mL^−1^) were inoculated to intact pepper fruits and incubated in a humid box for 2 days, respectively. The CO and DS indicate conidium and dendroid structure, respectively. (**E**) Quantitative measurement of penetration. The penetration was evaluated in at least 100 conidia and calculated the proportion of conidia that induced dendroid structure in a cuticle layer of pepper fruit. (**A and D**) Scale bar = 10  µm. (**C and E**) A significant difference was estimated by Duncan’s test (*P* < 0.05) and indicated by different lowercase letters.

*ΔCsczf12/crz1* showed defects in mycelial growth, conidiation, morphology, germination of conidium, and appressorium formation compared to wild-type and *Cscrz1c* strains (Table 1; Fig. 3A). Moreover, it was unable to grow on potato dextrose agar (PDA) containing 0.2 M CaCl_2_ and its mycelial growth was significantly inhibited on PDA containing 300 ppm Congo red compared to wild type and *Csczf12/crz1c* (Fig. S5A). These results suggest that *CsCZF12/CRZ1* is involved in fungal growth, differentiation, and stress adaptions of *C. scovillei*. When inoculated onto wounded pepper fruits, *ΔCsczf12/crz1* showed significantly reduced pathogenicity compared to wild-type and *Csczf12/crz1* strains (Fig. 3B and C). When inoculated onto intact pepper fruits, it caused only small sunken spots, whereas both wild type and *Csczf12/crz1c* showed induction of typical anthracnose disease (Fig. 3B and C). Microscopic examination showed that 91.2% ± 2.6% and 90.9% ± 2.5% of conidia of wild type and *Csczf12/crz1c*, respectively, formed appressoria and induced dendroid structures in the cuticle layer of intact pepper fruits after 2 days (Fig. 3D and E). However, only 11.2% ± 2.2% of *ΔCsczf12/crz1* conidia formed appressoria and induced dendroid structures, which were smaller in size than those induced by wild-type conidia (Fig. 3D and E). These results suggest that *CsCZF12/CRZ1* plays important roles in cuticle penetration and invasive hyphal growth of *C. scovillei*.

### *CsCZF12/CRZ1* is associated with cell survival and autophagy

*ΔCsczf12/crz1* was defective in conidium viability compared to wild-type and *Csczf12c/crz1c* stains (Fig. S5B). Autolysis of aerial hyphae occurred in *ΔCsczf12/crz1* after 5 days of culture on oatmeal agar (OMA), which was not observed in the wild type or *Csczf12/crz1c* (Fig. S5C). Unlike the wild-type or *Csczf12/crz1c* strain, conidiation in *ΔCsczf12/crz1* continuously decreased over a prolonged culture period (Fig. S5D). These results suggest that *CsCZF12/CRZ1* may be involved in cellular survival in response to nutritional limitations.

**Fig 4:**
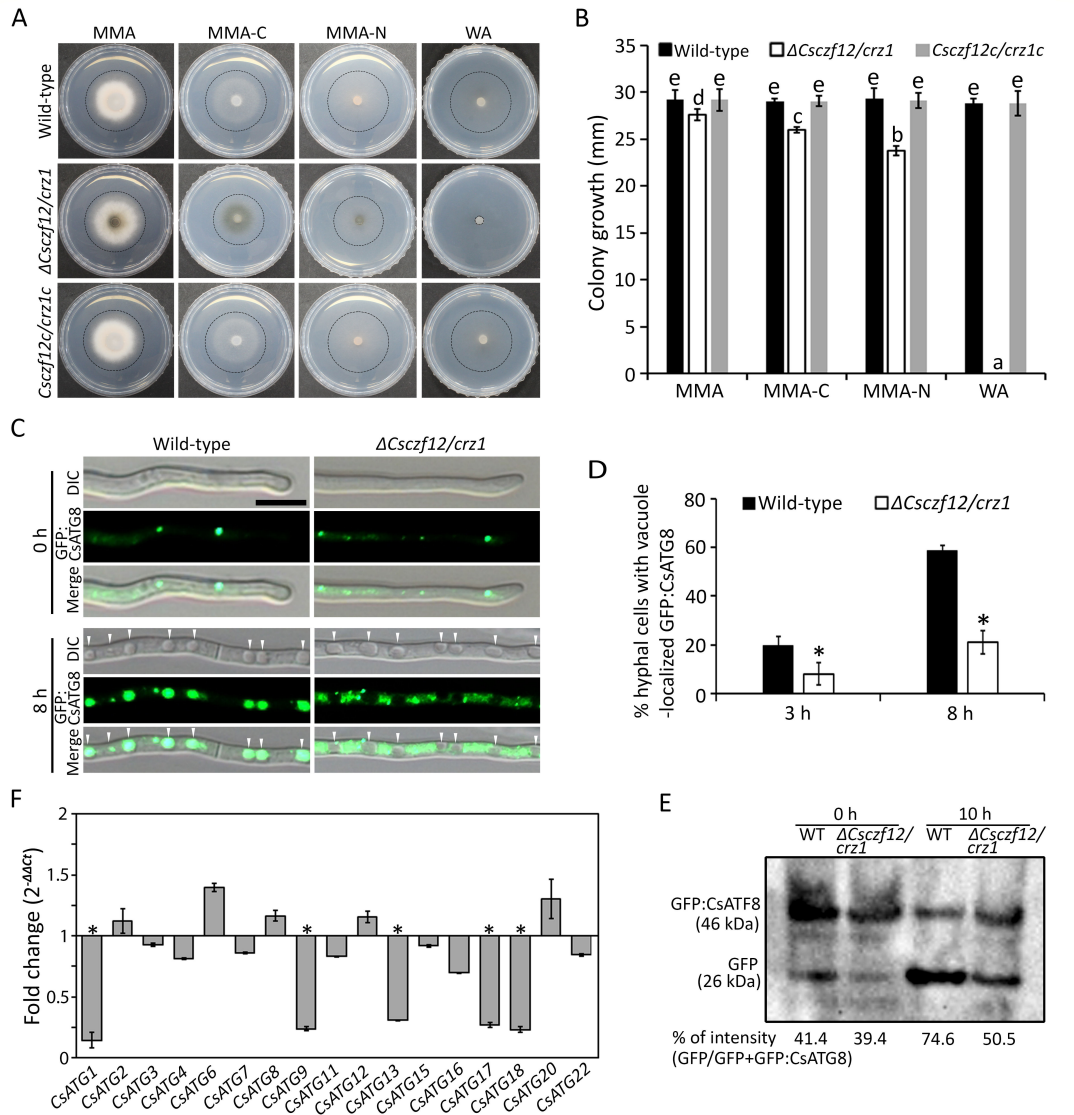
The *CsCZF12/CRZ1* is involved in tolerance to starvation and regulation of autophagy. (**A and B**) Mycelial growth under starvation conditions. Mycelial agar plugs (5 mm in diameter) from 3-day-old MMA cultures were inoculated to MMA, MMA-C, MMA-N, and water agar and incubated at 25°C and darkness for 5 days. (**A**) Photographs of mycelial growth. The dashed circular lines on photographs indicate the margin of colony growth. (**B**) Quantitative measurements of mycelial growth. The mycelial growth rate was determined by measuring colony diameters. A significant difference in a group was estimated by Duncan’s test (*P* < 0.05) and indicated by different lowercase letters. (**C and D**) Evaluation of vacuole-localized GFP:CsATG8. Mycelia of wild-type and *ΔCscrz1* strains expressing GFP:CsATG8 were cultured in CM broth, washed twice with sterilized distilled water, and shaken in MM-N for 8 h. (**C**) Visualization of GFP:CsATG8 in mycelia. The white triangles indicate vacuoles. Scale bar = 10 µm. (**D**) Evaluation of cells with vacuole localized GFP:CsATG8. At least 50 hyphal cells were examined per replicate. A significant difference in a group was estimated by Duncan’s test (*P* < 0.05) and indicated by an asterisk (*). (**E**) Immunoblot analysis of GFP:CsATG8 using anti-GFP antibody. Mycelia of the wild-type and *ΔCscrz1* strains expressing GFP:CsATG8 were shaken in MM-N for 0 and 10 h. The autophagy was evaluated by calculating the percentage of the free GFP in the total amount of free GFP and GFP:CsATG8. The intensity of protein bands in Western blot was measured using Image J. (**F**) Expression of autophagy-related genes (ATGs) in *ΔCsczf12/crz1* compared to the wild-type strain. Total RNA was extracted in mycelia of wild-type *ΔCscrz1*, which were grown in CM broth then transferred to MM-N and cultured for 2 h. The asterisks (*) indicate that the gene expressions were differentially downregulated (fold change <0.5).

Next, we evaluated mycelial growth under conditions of carbon and nitrogen starvation. *ΔCsczf12/crz1* failed to grow on water agar and showed significantly reduced mycelial growth compared to the wild-type or *Csczf12/crz1c* strains on minimal medium agar (MMA) minus a carbon source (MMA-C) and minus a nitrogen source (MMA-N) (Fig. 4A and B). These observations suggest that *CsCZF12/CRZ1* is involved in the tolerance of *C. scovillei* to starvation stress. In filamentous fungi, autophagy is associated with cellular survival and tolerance to nutritional depletion, such as carbon and nitrogen starvation (30, 31). To examine whether *CsCZF12/CRZ1* is involved in the regulation of autophagy, GFP was fused to the N-terminus of CsATG8, an ATG8 homolog, which indicates autophagic flux (32, 33). Both the wild-type and *ΔCsczf12/crz1* strains expressing GFP:CsATG8 fusion protein showed punctate localization of fluorescence in conidia and appressoria (Fig. S5E). However, *ΔCsczf12/crz1* showed significantly less accumulation of fluorescent signal in conidia and appressoria than the wild-type strain (Fig. S5E). Following nitrogen starvation (MM-N), *ΔCsczf12/crz1* showed significantly less vacuolar localization of CsATG8:GFP in mycelia (3 h, 8.1% ± 2.3%; 8 h, 20.9% ± 4.8%) than the wild-type strain (3 h, 19.7% ± 4.0%; 8 h, 58.6% ± 4.4%) (Fig. 4C and D). Immunoblotting assay showed that the level of autophagy was markedly decreased in *ΔCsczf12/crz1* compared to the wild-type strain (Fig. 4E). The expression levels of 5 of 17 autophagy-related genes (ATGs) were downregulated in *ΔCsczf12/crz1* compared to wild type under MM-N conditions (Fig. 4F). These results suggest that *CsCZF12/CRZ1* is involved in regulating autophagy of *C. scovillei*.

### *CsCZF9* is essential for appressorium formation and pathogenicity

**Fig 5:**
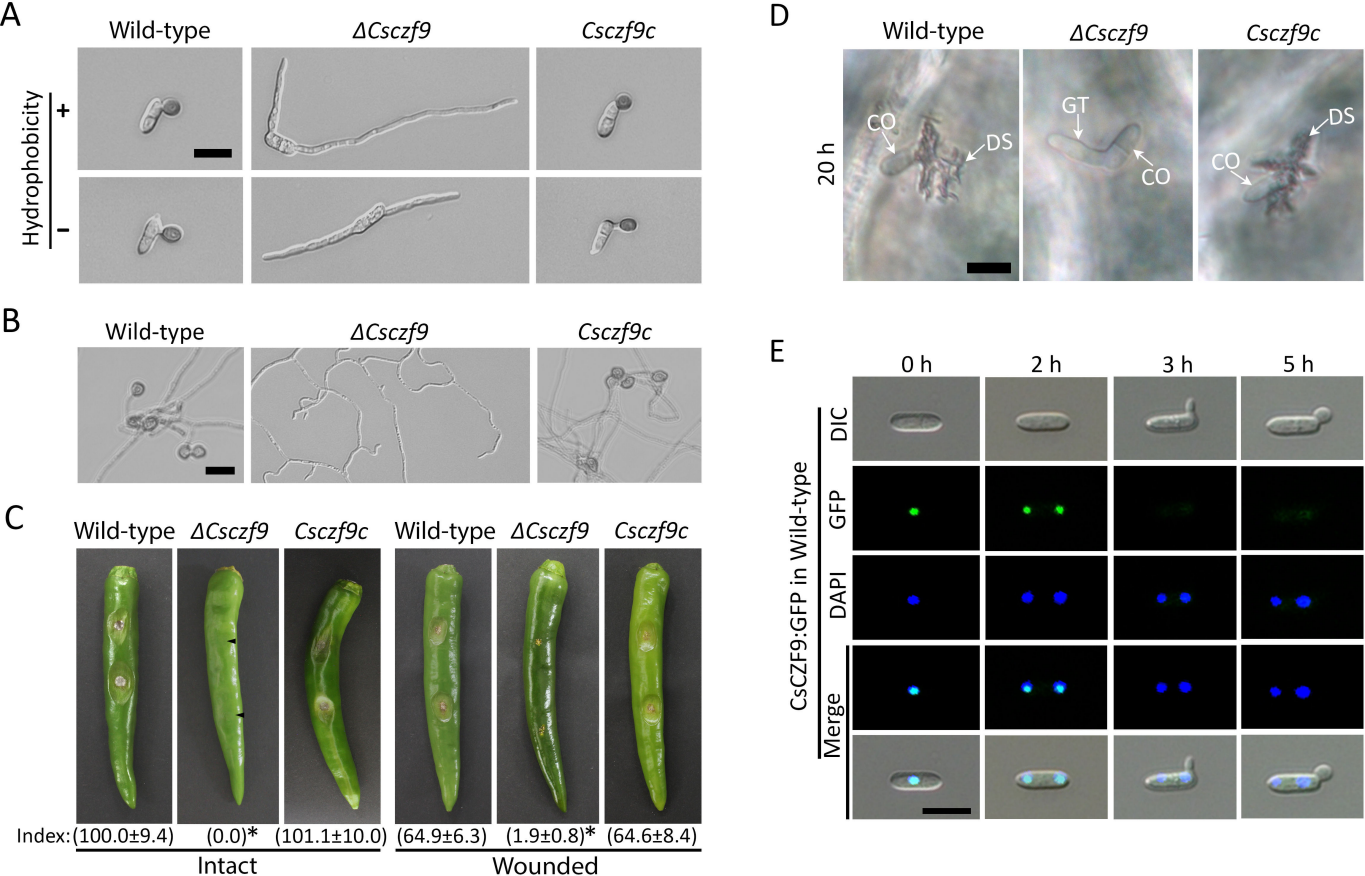
The *CsCZF9* is critical for appressorium formation and pathogenicity and localized in nuclei. (**A**) Appressorium formation. Drops of conidial suspensions (5 × 10^4^ mL^−1^) were placed on the hydrophobic coverslips and hydrophilic slide glasses and incubated in a humid box at 25°C for 16 h. (**B**) Appressorium-like structure formation. Mycelial agar plugs from 3-day-old OMA were placed on the hydrophobic coverslips and incubated in a humid box at 25°C for 5 days. (**C**) Pathogenicity assay. Conidial suspensions (2.5 × 10^5^ mL^−1^) were inoculated to wounded and intact pepper fruits and incubated in humid boxes for 6 and 8 days, respectively. The lesion size was measured using Image J and normalized to that on intact pepper fruits caused by the wild-type strain as a relative of 100%. The values of relative lesion size are shown below in photographs. A significant difference was estimated by Duncan’s test (*P* < 0.05) and indicated by asterisks (*). (**D**) Visualization of appressorium-mediated penetration. Conidial suspensions (5 × 10^4^ mL^−1^) were inoculated to intact pepper fruits and incubated in humid boxes for 20 h. CO, GT, and DS indicates conidium, germ tube, and dendroid structure, respectively. (**E**) Subcellular localization of CsCZF9. Conidial suspensions (5 × 10^4^ mL^−1^) from transformants expressing CsCZF9:GFP fusion protein were dropped to the hydrophobic coverslip and incubated in a humid box at 25°C. The nuclei were stained with DAPI. (A, B, and D) Scale bar = 10 µm.

*ΔCsczf9* produced significantly fewer conidia than wild-type and *Csczf9c* strains (Table 1), suggesting that *CsCZF9* is involved in the conidiation of *C. scovillei*. Strikingly, conidia produced by *ΔCsczf9* were able to germinate but failed to develop appressoria on hydrophobic and hydrophilic surfaces (Fig. 5A). Moreover, its hyphal tips were unable to differentiate appressorium-like structures (ALSs) on hydrophobic surfaces (Fig. 5B). The germ tube and hyphal tip were straight and non-swollen in *ΔCsczf9* (Fig. 5A and B). However, both wild-type and *Csczf9c* strains developed appressoria and ALSs on such inductive surfaces (Fig. 5A and B). These results suggest that *CsCZF9* is essential for surface recognition to allow appressorium formation of *C. scovillei*. When inoculated onto intact pepper fruits, both wild type and *Csczf9c* developed anthracnose disease, whereas *ΔCsczf9* was completely nonpathogenic (Fig. 5C). Microscopic examination showed that *ΔCsczf9* failed to form appressoria on intact pepper fruits (Fig. 5D). When inoculated onto wounded pepper fruits, *ΔCsczf9* showed marked pathogenicity compared to wild-type and *Csczf9c* (Fig. 5C). These results suggest that *CsCZF9* is important for the development of anthracnose disease on pepper fruits. Expression of the CsCZF9:GFP fusion protein in the wild-type strain showed that CsCZF9 was localized in the nuclei of ungerminated conidia in response to hydrophobic surfaces (Fig. 5E).

### *CsCZF9* is important for the suppression of plant defense mechanisms

**Fig 6:**
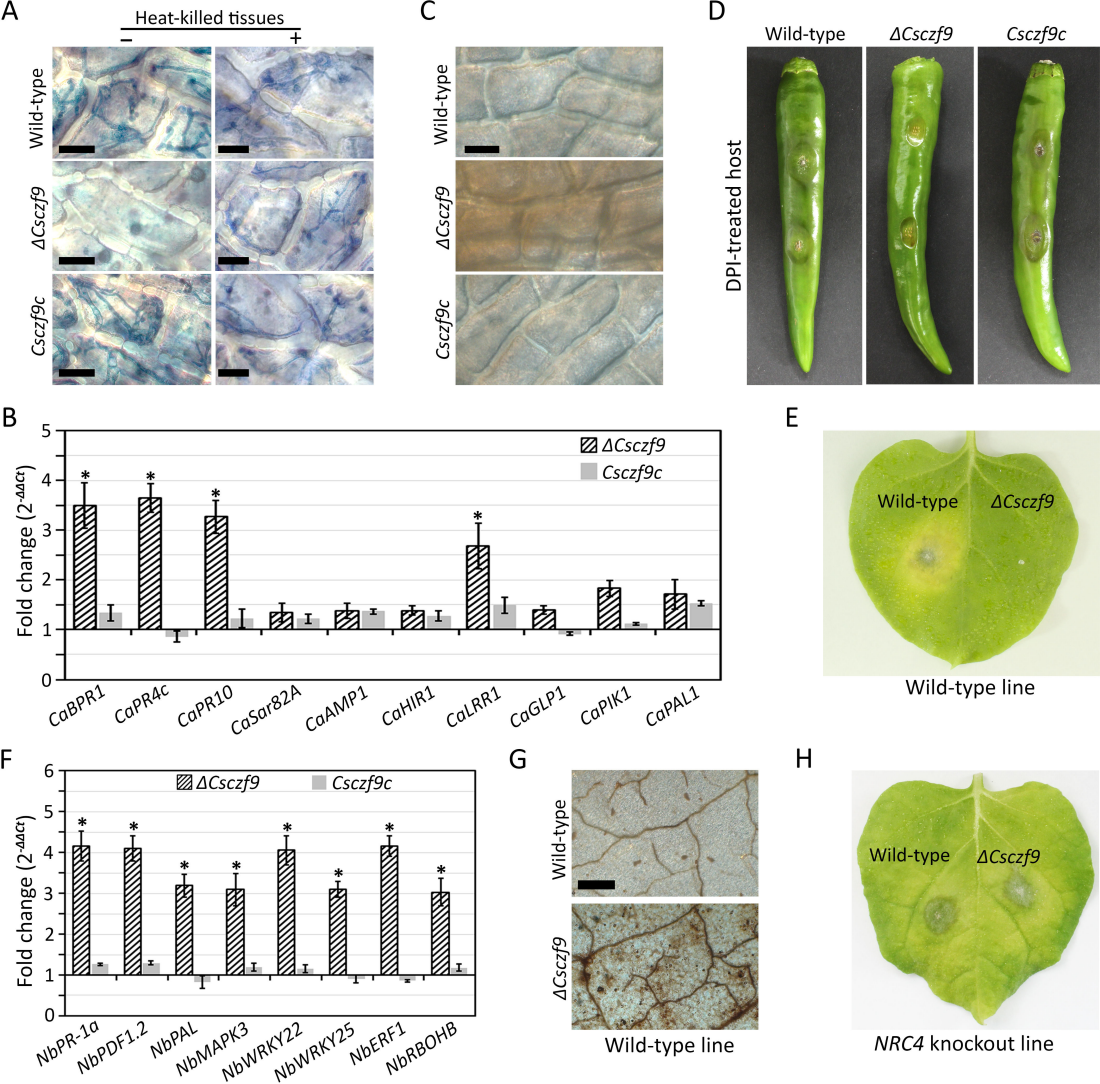
The *CsCZF9* is involved in the suppression of plant defense mechanisms. (**A**) Visualization of invasive growth. Conidial suspensions (5 × 10^4^ mL^−1^) were inoculated to living and heat-killed tissues with artificial wound of pepper fruit and incubated in a humid box for 5 and 3 days, respectively. Invasive hyphae were stained with blue color using a trypan blue staining. Scale bar = 20 µm. (**B**) Expression of host defense-related genes in pepper fruit infected with the *ΔCsczf9* strain, compared to the wild-type and *Csczf9c* strains. Total RNA was extracted 2 days after inoculating conidial suspensions (5 × 10^5^ mL^−1^) to wounded pepper fruit. The pepper actin gene was used as a reference. The asterisks (*) indicate that the gene expressions were differentially upregulated (fold change >2). (**C**) Staining of ROS in infected pepper fruits. Conidial suspensions (5 × 10^4^ mL^−1^) were dropped to wounded pepper fruit and incubated in a humid box for 3 days. Thin sections near the infected sites were cut and immersed in a DAB solution (1  mg mL^−1^ [pH 7.5]) and boiled in 95% (vol/vol) ethanol for 10  min. Scale bar = 30 µm. (**D**) Anthracnose disease formation on DPI-treated pepper fruits. Conidial suspensions (5 × 10^5^ mL^−1^) were inoculated to wounded pepper fruit, which were priorly treated with a DPI solution (2.5  µM) and incubated in a humid box. Photographs were taken after 6 days. (**E and H**) Infections of wild-type and *ΔCsczf9* strains on *N. benthamiana* plants. Leaves of 8-week-old wild-type (**E**) and *NRC4* knockout (**H**) *N. benthamiana* plants with prior artificial wounds were inoculated with mycelial agar plugs (2 mm in length) obtained from 3-day old water agar cultures and incubated in a humid box for 5 days. (**F**) Expression of host defense-related genes in *N. benthamiana* leaves infected with the *ΔCsczf9* strain, compared to the wild-type and *Csczf9c* strains. Total RNA was extracted at 2 days after inoculation of hyphal agar plugs to wounded leaves of wild-type *N. benthamiana* plant. The *N. benthamiana* actin gene was used as a reference. The asterisks (*) indicate that the gene expressions were differentially upregulated (fold change >2). (**G**) Staining of ROS in infected leaves of *N. benthamiana* plant. Leaves of wild-type *N. benthamiana* were inoculated with hyphal agar plugs. After 3 days, discs of infected leaves were immersed in a DAB solution (1  mg mL^−1^ [pH 7.5]) and boiled in 95% (vol/vol) ethanol for 30  min. Scale bar = 300 µm.

*ΔCsczf9* is defective in invasive hyphal growth in wounded fruits, which was restored when inoculated onto heat-killed tissues of wounded pepper fruits (Fig. 6A), suggesting that *CsCZF9* may be involved in the suppression of host defense mechanisms. Therefore, we evaluated the expression of pepper defense-related genes by qRT-PCR. These genes, including *CaBPR1*, *CaPR4c*, *CaPR10*, and *CaLRR1* (9), were highly upregulated in wounded pepper fruits inoculated with *ΔCsczf9* compared to the wild-type and *Csczf9c* strains (Fig. 6B). To determine whether *CsCZF9* is involved in suppression of reactive oxygen species (ROS) burst, we conducted staining with 3,3′-diaminobenzidine (DAB) and found marked H_2_O_2_ accumulation in pepper epidermal cells infected with *ΔCsczf9* but not in those of the wild-type strain (Fig. 6C). Infectivity of *ΔCsczf9* was partially recovered by treating wounded pepper fruits with diphenyleneiodonium (DPI), an inhibitor of ROS generation (Fig. 6D). As several *Colletotrichum* species, including *Colletotrichum orbiculare*, *Colletotrichum capsici*, and *Colletotrichum acutatum*, infect the leaves of *Nicotiana benthamiana*, a widely used model plant (34–37), we inoculated wild-type and *ΔCsczf9* strains onto leaves of wild-type *N. benthamiana* plants. The wild-type strain, but not *ΔCsczf9*, produced visible lesions on wounded leaves of wild-type *N. benthamiana* plants (Fig. 6E; Fig. S6), suggesting that *CsCZF9* is required for the pathogenicity of *C. scovillei* on *N. benthamiana*. Next, we evaluated the expression of genes related to plant defense and found that *NbPR-1a*, *NbPDF1.2*, *NbPAL*, *NbMAPK3*, *NbWRKY22*, *NbWRKY25*, *NbERF1*, and *NbRBOHB* were highly upregulated in leaves of wild-type *N. benthamiana* plants inoculated with *ΔCsczf9* compared to the wild-type strain (Fig. 6F; Table S3). DAB staining revealed a marked accumulation of H_2_O_2_ in leaves of wild-type *N. benthamiana* infected with *ΔCsczf9* but not the wild-type strain (Fig. 6G). These results suggest that *CsCZF9* is involved in the suppression of defense responses in *N. benthamiana*. Therefore, we examined whether *ΔCsczf9* is capable of infecting the leaves of immunocompromised mutant *N. benthamiana* plants lacking the *NRC4* gene. *NRC4* has been shown to encode a helper leucine-rich repeat-containing (NLR) protein, which functions with multiple sensor NLRs in a receptor network and is important for plant defense upon pathogen attack (38, 39). The results showed that *ΔCsczf9-*infected leaves of *NRC4* knockout *N. benthamiana* plants were similar to the wild-type strain (Fig. 6H). These results suggest that *CsCZF9* is important for fungal pathogenicity and suppression of plant defenses by *C. scovillei*.

### CsCZF9 is a novel target of CsPMK1

**Fig 7:**
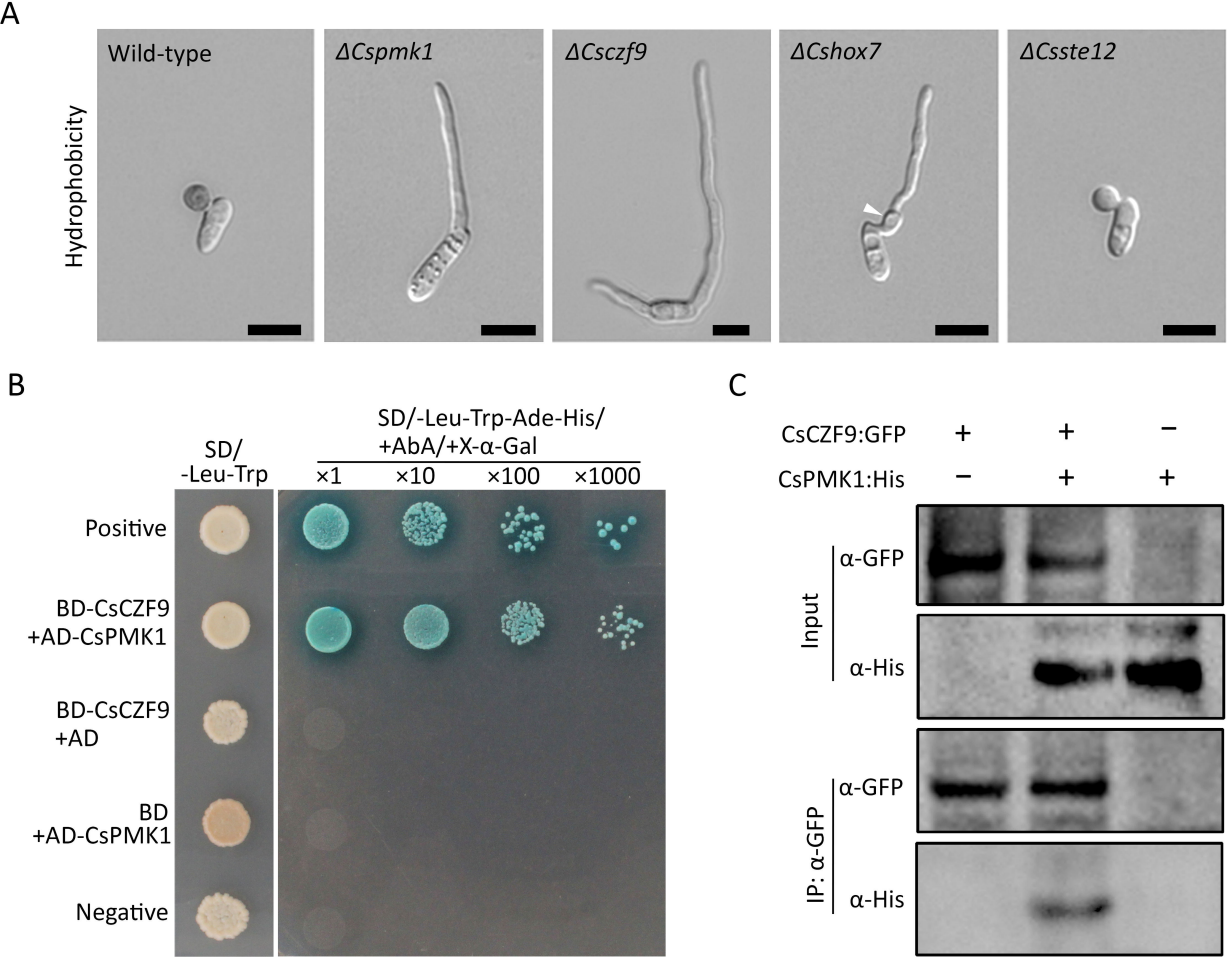
Visualization of appressorium formation of deletion mutants and interaction between CsPMK1 and CsCZF9. (**A**) Appressorium formation. Conidial suspensions (5 × 10^4^ mL^−1^) obtained from 7-day-old OMA were dropped onto the hydrophobic coverslip and incubated in a humid box at 25°C. The white triangle indicates swelling in the germ tube. Scale bar = 10 µm. (**B**) Y2H assay. The yeast transformants expressing pGBKT7-53 and pGADT7-T (positive control), pGBKT7-CsCZF9 and pGADT7-CsPMK1, pGBKT7-CsCZF9 and pGADT7, pGBKT7 and pGADT7-CsPMK1, and pGBKT7-Lam and pGADT7-T (negative control) were grown on SD-Leu-Trp and SD/+Aureobasidin A/+ X-α-Gal/−/−Leu-Trp-Ade-His. (**C**) Co-immunoprecipitation assay. Conidia of the wild-type strain expressing CsCZF9:GFP, CsPMK1:His, and both CsCZF9:GFP and CsPMK1:His were harvested from 7-day-old OMA and used for total protein extraction. Proteins eluted from anti-GFP agarose beads were detected with anti-His and anti-GFP antibodies.

In the blast fungus *Magnaporthe oryzae,* appressorium development is mainly regulated by two Pmk1 targets, that is, MoHOX7 and Mst12 (40, 41). In *C. scovillei*, *ΔCshox7* is defective in appressorium formation but not surface recognition (9). In our study, deletion of the *Mst12* ortholog in *C. scovillei* yielded a mutant (*ΔCsste12*) with defective appressorium maturation and penetration (Fig. 7A; Fig. S7A through E). *ΔCsczf9* was defective in surface recognition to allow appressorium formation and suppression of host defense responses (Fig. 5A through D and 6A through H), resembling the phenotype of *ΔCspmk1* (15). To examine whether CsCZF9 interacts with CsPMK1, yeast two-hybridization (Y2H) analysis was performed. The transformants expressing pGADT7-CsPMK1 and pGBKT7-CsCZF9 grew on both double dropout medium (DDO) and quadruple dropout medium (QDO), as did transformants expressing positive control vectors (Fig. 7B). However, transformants expressing negative control vectors failed to grow on QDO. These observations suggest that CsCZF9 interacts with CsPMK1 in yeast. To confirm the interaction between CsCZF9 and CsPMK1, the recombinant construct CsPMK1:His was expressed in both the wild-type strain and wild-type strains expressing CsCZF9:GFP. Total proteins extracted from wild-type expressing CsCZF9:GFP, CsPMK1:His, and both CsCZF9:GFP and CsPMK1:His were immunoprecipitated with anti-GFP agarose. After immunoprecipitation, only proteins from the wild-type strain expressing both CsCZF9:GFP and CsPMK1:His could be detected with anti-His antibody in Western blotting analysis (Fig. 7C). These observations suggest that CsCZF9 interacts with CsPMK1 in *C. scovillei*. Next, a phosphorylation assay was performed because the amino acid sequence of CsCZF9 was predicted (by the online tool NetPhos 3.1) to contain four putative MAPK phosphorylation sites (Table S4). The result showed that the level of phosphorylated CsCZF9 was lower in *ΔCspmk1* than in the wild-type strain (Fig. S8), suggesting that CsCZF9 is phosphorylated by CsPMK1.

## DISCUSSION

**Fig 8:**
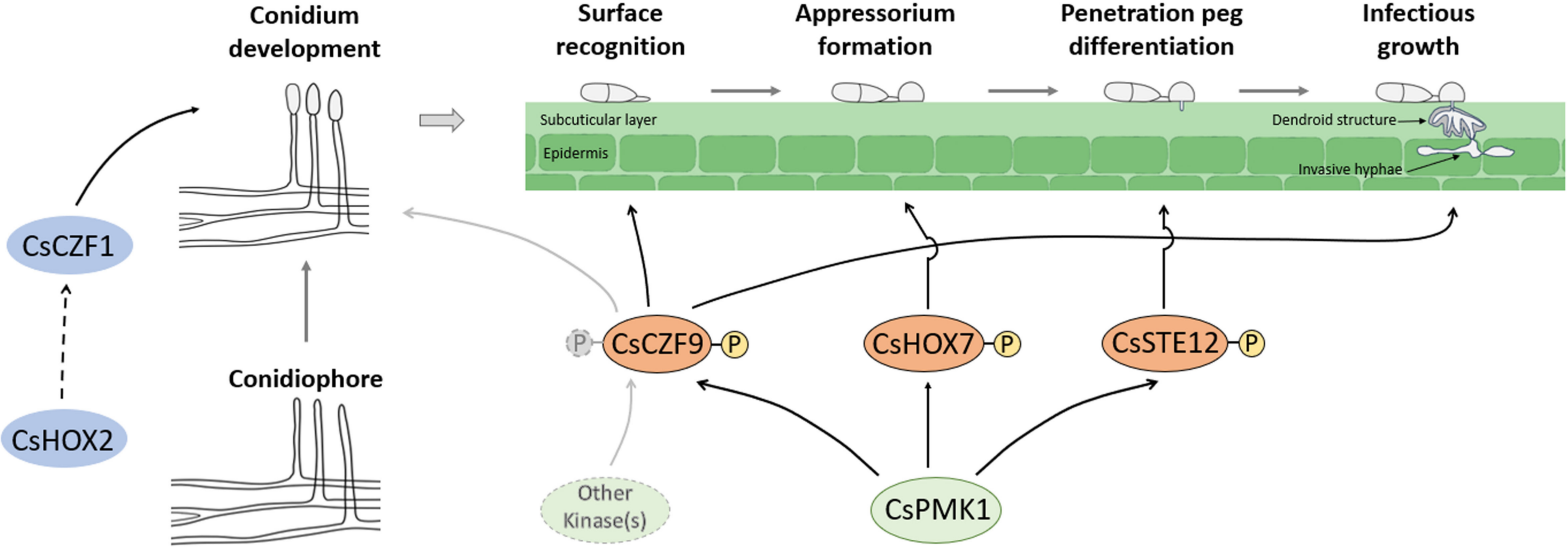
A proposed a model for the putative mechanisms of the CsHOX2-mediated conidiation and the MAPK CsPMK1-mediated appressorium infection in *C. scovillei*. The CsHOX2 positively regulates the expression of CsCZF1 to control conidium reproduction. In response to inductive signals, the CsPMK1 is activated and enters to nuclei of conidia. The nuclear-localized CsPMK1 phosphorylates several TFs, including CsCZF9, CsHOX7, and CsSTE12 to control surface recognition for appressorium initiation, appressorium formation, and appressorium penetration, respectively. After cuticle penetration, other unknown kinases phosphorylate CsCZF9 to suppress defense-related mechanisms to promote infection of *C. scovillei*.

The *CZFs* play crucial roles in molecular events that are important for fungal biology and pathogenicity (22–28). Our findings show that nine *CsCZFs* are involved in the conidiation of *C. scovillei*, in which *CsCZF1* is a stage-specific regulator of conidiation (Table 1; Fig. 1A through E). *ΔCsczf1* was defective only in conidium reproduction from the conidiophore, similar to the same phenotype of the previously reported deletion mutant *ΔCshox2* (9). *CsHOX2* orthologs have been shown to regulate conidiation in some fungal pathogens (9, 40, 42, 43). As expected, we found that *CsCZF1* expression was markedly reduced in *ΔCshox2* and the constitutive expression of *CsCZF1* partially recovered conidiation in *ΔCshox2* (Fig. 2B through D). This suggests that *CsCZF1* functions downstream of *CsHOX2* to control conidiation in *C. scovillei* (Fig. 8). Unlike *CsCZF1*, four other genes (*CsCZF3*, *CsCZF8*, *CsCZF10*, and *CsCZF12/CRZ1*) were involved in both conidiation and conidium morphology (Table 1). Conidia produced by *ΔCsczf3*, *ΔCsczf8*, and *ΔCsczf10* were larger in size and showed impaired viability and conidium-mediated anthracnose development under the condition of heat shock. We showed previously that four *CsHOX* deletion mutants produced large conidia, which were defective in stress tolerance and conidium development (9). Abnormally large conidia exhibited increased sensitivity to ambient environmental conditions, reflecting not only a simple morphological change but also a metabolic disorder and disadvantages in dissemination *via* air currents in *C. scovillei*.

CsCZF12/CRZ1 (orthologous to Ca^2+^/calmodulin-responsive transcription factor Crz1) is involved in mycelial growth, conidiation, conidium morphology, appressorium formation, and plant infection of *C. scovillei* (Table 1; Fig. 3A through E). These findings are consistent with its ortholog, CgCrzA, in *Colletotrichum gloeosporioides* (25). Unlike other fungal plant pathogens, CsCZF12/CRZ1 has been shown to regulate autophagy in *C. scovillei* (25, 44, 45). In our study, this was evidenced by the defective mycelial growth phenotype of *ΔCsczf12/crz1* under conditions of carbon and nitrogen starvation (Fig. 4A and B), along with reduced autophagosome formation in conidia and appressoria (Fig. S5E), reduced autophagic flux in mycelia under conditions of nitrogen starvation (Fig. 4C through E), and downregulated expression of autophagy-related genes compared to wild type under conditions of nitrogen starvation (Fig. 4F). Autophagy is a conserved process that is important for supporting survival *via* the recycling of unnecessary organelles and proteins (33, 46). Autophagy is transcriptionally regulated by several TFs from the fork-head box, zinc finger, and basic helix-loop-helix leucine TF families (47–49). Unlike plant pathogenic fungi, in the opportunistic fungal pathogen *Aspergillus fumigatus*, Crz1 positively regulates autophagy by positively modulating the expression of the *Atg9* ortholog (50). In our study, deletion of *CsCZF12/CRZ1* caused multiple impairments of fungal differentiation, tolerance to cell wall integrity and calcium stresses, and pathogenicity of *C. scovillei*. These defects in *ΔCsczf12/crz1* would be partially due to its defect in autophagy, as autophagy plays a role in the degradation and recycling of cellular components to maintain cellular homeostasis in fungal pathogens, thus contributing to nutrient limitation, adaptation to the host environment, and intracellular invasion within plant cells.

Strikingly, we found that *CsCZF9* was a novel early regulator essential for surface recognition to allow appressorium formation in association with Pmk1 in *C. scovillei*. We previously confirmed that the CsPMK1 protein, which is expressed in the nuclei of conidia and appressoria, is involved in sequential events during appressorium development from recognizing the host surface for initiating appressorium development to functional maturation of the appressorium (15). In the present study, we found that CsPMK1 interacted with and phosphorylated CsCZF9, functioning as a downstream target of CsPMK1 to control appressorium initiation in *C. scovillei* (Fig. 7A through C; Fig. S8). Consistent with these observations, CsCZF9 protein began to be expressed in the nuclei of ungerminated conidia (Fig. 5E). In the course of appressorium development, CsPMK1 also regulates the homeobox TF CsHOX7, which is involved in appressorium formation but not in surface recognition in *C. scovillei* expressed in the nuclei of conidia developing appressoria (9). In addition, we also showed that *CsSTE12*, which is orthologous to *Ste12* and encodes another downstream target of Pmk1, plays an essential role in appressorium maturation and penetration (Fig. S7A through E). Taken together, our findings indicate that the three TF targets are key stage-specific regulators phosphorylated by CsPMK1 protein for appressorium development in *C. scovillei* (Fig. 8).

In addition to its critical function in appressorium initiation, *CsCZF9* plays an essential role in invasive hyphal growth in host cells. Wounded pepper fruit inoculated with *ΔCsczf9* accumulated ROS and upregulated genes related to pepper defense (Fig. 6A through C). *ΔCsczf9* showed recovery of invasive hyphal growth in heat-killed tissues and anthracnose lesions on DPI-treated wounded pepper fruits (Fig. 6A and D). Furthermore, it showed defective infection, suppression of ROS generation, and the expression of defense-related genes when inoculated onto leaves of wild-type *N. benthamiana* plants (Fig. 6E through H). However, it recovered infectivity when inoculated onto leaves of *NRC4* gene knockout *N. benthamiana* plants with compromised host defense mechanisms (38, 51). These findings suggest a crucial role of *CsCZF9* in the suppression of host defense responses of *C. scovillei*. We previously showed that *ΔCspmk1* fails to grow invasive hyphae due to a defect in suppression of host defense responses, whereas *ΔCshox7* and *ΔCsste12* show normal infective growth (9, 15). Therefore, it is reasonable to suggest that *CsCZF9* suppresses the host defense response under the control of CsPMK1 in *C. scovillei* (Fig. 8).

It is not surprising that the conidiation of *C. scovillei* was inhibited by the deletion of *CsCZF9* but not *CsPMK1*. As described previously in *M. oryzae*, Pmk1 shares a mix of functional similarities and differences with two of its target proteins, that is, PIC1 and PIC5 (20). Furthermore, many TFs are regulated by posttranslational modifications, particularly phosphorylation/dephosphorylation, in response to external stimuli (40, 52). Multiple phosphorylation sites within a TF would function as a signal modulator, with altered phosphorylation resulting in changes in the amplitude of gene expression (52). The results of *in silico* analysis indicated the presence of a dozen putative phosphorylation sites within the sequence of CsCZF9 targeted by other protein kinases, including protein kinase C, casein kinase I and II, and cyclin-dependent kinase 2. These kinases have been implicated in sexual and asexual reproduction in fungal pathogens (53–55). It is possible that the CsCZF9 protein with the modification of phosphorylation may have different DNA-binding affinity to *cis*-elements and other TFs important for fungal development (Fig. 8). Further studies are required to investigate these issues.

*C. scovillei* employs a subcuticular-intracellular lifestyle to infect pepper fruits. This lifestyle has been reported in other pathosystems, such as the *Colletotrichum lupini*-lupin stem and *C. gloeosporioides*-tomato fruit interactions (Fig. S9) (56, 57). The subcuticular intramural hyphae of *C. lupini* formed in the subcuticular layer of the lupin stem are distinct, being swollen and unbranched (56), whereas *C. scovillei* develops highly branched hyphae, visualized as dendroid structure in a subcuticular layer of pepper fruit (Fig. S9). *C. gloeosporioides* develops highly branched hyphae in the subcuticular layer and undergoes the quiescent stage before the maturity of tomato fruit (57), which is different from *C. scovillei*-pepper fruit interaction (Fig. S9). These represent that *Colletotrichum* species have evolved diverse infection strategies on different hosts. Our study provides novel insights into the roles of C_2_H_2_ zinc finger TFs in conidiation, autophagy, and appressorium-mediated plant infection in *C. scovillei*. The findings contribute to our understanding of the comprehensive networks governing the occurrence and dissemination of pepper fruit anthracnose disease, with potential benefits in developing novel strategies to control anthracnose disease on economically important fruits.

## MATERIALS AND METHODS

### Culture conditions, data availability, and bioinformatic tools

The *C. scovillei* strain KC05 was used as the wild-type strain in this study (15). The V8 agar (V8A), PDA, OMA, and MMA were used to routinely grow the wild type and its transformants (9). Fungal tissues for nucleic acid extraction were cultured in broth of complete medium (CM) and on TB3 agar (10). The genomic data of *C. scovillei* strain KC05 have been deposited in FigShare (https://doi.org/10.6084/m9.figshare.26112565.v1). Genomic sequences of other fungal species were downloaded from GenBank (https://www.ncbi.nlm.nih.gov/genbank/). The CZFs of *C. scovillei* and other fungal species were isolated by predicting their domain using InterProScan (https://www.ebi.ac.uk/interpro). Phylogenetic tree was generated using MEGA X and sequences were illustrated using BioEdit 7.2.

### Generation of transformants

To generate gene knockout transformants, the deletion constructs prepared by a modified double-joint PCR were introduced to the wild-type protoplast *via* a previously reported (7, 9, 58). For complementation, the sequences of each gene containing the promoter, open reading frame (ORF), terminator, and a 1.5 kb Geneticin resistance cassette were co-transformed into protoplasts of desired deletion mutants (15). The deletion mutants and complemented strains were verified *via* Southern blotting and RT-PCR (59). To perform subcellular localization, the fragments containing the promoter and ORF of *CsCZF1* minus stop codon were amplified with primers CAP_012989.1 5 F/CAP_012989.1 R (Table S5). Fragments of green fluorescent protein (GFP) were amplified with primers CAP_012989.1 _ GFP F/GFP NR (Table S5). The GFP sequence was fused to the C terminus of *CsCZF1* and was further transformed into wild-type protoplasts. Localizations of CsCZF9 and CsSTE12 were performed according to the same method. To generate N-terminal fusion of GFP to CsATG8, the sequence of *CsATG8* promoter, GFP, CsATG8 ORF, and CsATG8 ORF terminator were amplified with primers CsATG8 F/CsATG8_Pro_R, CsATG8_Pro_GFP F/GFP_CsATG8 R, and CsATG8_ORF F/CsATG8 R, respectively (Table S5). The amplified sequences were fused to generate recombinant construct Pro^CsATG8^:GFP:CsATG8, which were further transformed into wild-type and *ΔCscrz1* protoplasts. To generate constitutive expression construct of *CsCZF1*, promoter of *Neurospora crassa isocitrate lyase* gene was amplified with primers ICL F/ICL R (Table S5). The sequence of ORF and terminator of CsCZF1 was amplified with primers ICL_CAP_012989.1 F/CAP_012989.1 3R (Table S5). Two amplified fragments were fused and transformed into *ΔCshox2* protoplasts. To generate C-terminal fusion of 6 × His to CsPMK1, a sequence containing promoter and ORF of CsPMK1 was amplified with primers CsPMK1 5 F/CsPMK1_His R (Table S5). The sequence containing terminator of CsPMK1 was amplified with primers His_CsPMK1_Ter F/CsPMK1_3R (Table S5). Amplified sequences were fused and transformed protoplasts of wild-type strain expressing CsCZF9:GFP. All transformants were screened using PCR or checked the fluorescence signals using fluorescence microscopy (Carl Zeiss Microscope Division, Germany).

### Nucleic acid manipulation

Total RNA was isolated from frozen tissues of fungi and fungal infecting plant using an Easy-Spin Total RNA extraction kit (iNtRON Biotechnology, South Korea). The complementary DNA (cDNA) used for quantitative RT-PCR (qRT-PCR) was synthesized from 5 µg of total RNA using a SuperScript III first-strand synthesis kit (Invitrogen, CA, USA). The *C. scovillei β-tubulin (CAP_007327*) was used as an endogenous control gene in the qRT-PCR. The qRT-PCR was performed with two replicates in three independent experiments using a StepOne real-time PCR system (Applied Biosystems, CA, USA) and the relative transcripts were expressed as 2*^−^*^ΔΔCT^, with a *C. scovillei β-tubulin* gene as an endogenous control (10, 60). Genomic DNA was isolated using a quick and safe method for PCR and a standard method for Southern blotting (61, 62). To perform Southern blotting, the restriction enzyme-digested genomic DNA was separated in agarose gel, and transferred to a nylon membrane, which is probed with a DNA segment (about 500 bp), labeled with Biotin-High Prime (Roche, IN, USA).

### Characterization of phenotypes

Mycelial growth was evaluated by measuring the diameter of colony growth on PDA and V8 medium. Conidiation was determined by counting conidia harvested with 5 mL of sterilized distilled water (SDW) from 7-day-old V8 agar. To evaluate conidium morphology, the length and width of conidia from 7-day old OMA were measured. Drops of conidial suspension (5 × 10^4^ mL^−1^) were placed in the hydrophobic surface of coverslips and incubated in a humid plastic box. The conidial germination and appressorium formation were determined after 12 and 16 h, respectively. At least 100 conidia were examined per replicate in the evaluation of morphology and germination of conidium and appressorium formation. To perform pathogenicity assays, conidial suspensions (2.5 × 10^5^ mL^−1^) were dropped to the intact and pre-wounded pepper fruits and incubated in humid plastic boxes for 6 and 9 days, respectively. Data were collected from at least three independent experiments with three replicates per experiment. Significant differences were analyzed by Duncan’s test (*P* < 0.05).

### Yeast two-hybridization assay

The yeast transformation and assay for protein interaction were performed using the Matchmaker Gold Yeast Two-Hybrid System (Clontech, Mountain View, CA, USA) according to the manufacturer’s manual. The cDNA of *CsCZF9* was amplified with primers CAP_003653.1 _bait F/CAP_003653.1 _bait R and cloned into bait vector pGBKT7. The prey vector expressing CsPMK1 was generated previously (15). Both recombinant vectors were confirmed by sequencing and then co-introduced into competent cells of Y2H Gold strain (Clontech, Mountain View, CA, USA). Yeast transformants were screened on double dropout agar medium (SD-Leu-Trp) and quadruple dropout agar medium (SD-Leu-Trp-His-Ade/X-α-Gal/Aureobasidin A).

### Protein extraction and western blotting

Total protein was isolated using a phenol extraction method, modified from a previous protocol (63). Frozen fungal tissues were grinded into fine powder with liquid nitrogen. The powder (1.0 g) was mixed with 10 mL of the extraction buffer (0.7 M sucrose, 0.5 M Tris base, 30.0 mM HCl, 50.0 mM EDTA, 0.1 M KCl, 2% β-mercaptoethanol, and 2 mM phenylmethylsulfonylfluoride) and 10 mL of water-saturated phenol. After vigorously stirring for 30 min, the lysate was centrifuged at 12,000 rpm and 25°C for 10 min. The phenol phase of lysate was mixed with five volumes of methanol containing 0.1 M NH_4_Cl, next precipitated at −20°C for 2 h, and finally centrifuged at 12,000 rpm and 4°C for 15 min. The resulting pellet was washed with 1 mL of acetone pre-cooled at −20°C and centrifuge at 9,000 rpm and 4°C for 5 min. The eluted proteins were resolved in 8% SDS-PAGE and transferred to a PVDF membrane using the tank method at 110 V and 0°C for 1.5 h. The membrane was detected with anti-His-HRP (#46–0707, Invitrogen, CA, USA; 1: 4,000 dilution). The samples were also detected with anti-GFP Polyclonal Antibody (A-6455, Invitrogen, CA, USA; 1:8,000 dilution) and Goat Anti-Rabbit IgG H&L (HRP) (ab6721, Abcam, United Kingdom; 1:8,000 dilution).

### Co-immunoprecipitation and protein phosphorylation assays

Total protein was extracted from conidia of transformants expressing CsCZF9:GFP, CsCZF9:GFP, and both CsCZF9:GFP and CsPMK1:His as described above. The anti-GFP mAb Agaroses (Medical & Biological Laboratories Co., CA, USA) were individually added to each protein sample and slowly rotated at 4°C for 3 h. The anti-GFP mAb Agaroses bound proteins were spin down at 3,000 rpm and 4°C for 2 min, and then washed with elution buffer for four times. Eluted proteins were analyzed using western blot with anti-His and anti-GFP antibodies as described above. For protein phosphorylation assay, total protein was extracted from conidia of wild type and *CsPMK1* deletion mutant expressing CsCZF9:GFP. The protein samples were resolved in 10% SDS-polyacrylamide gels containing 150 µM ZnCl_2_ and 40 µM Phos-tag acrylamide AAL-107 (FUJIFILM Wako Pure Chemicals Co., Japan). The protein in Zn^2+^-phos-tag acrylamide gel was transferred to a PVDF membrane according to the manufacturer’s instructions. The membrane was detected with anti-GFP antibody as described above.

## SUPPLEMENTAL MATERIAL

Supplemental figures mbio.00667-24-s0001.pdf Fig. S1 to S9.

Supplemental tables mbio.00667-24-s0002.xlsx Tables S1 to S5.

ASM does not own the copyrights to Supplemental Material that may be linked to, or accessed through, an article. The authors have granted ASM a non-exclusive, world-wide license to publish the Supplemental Material files. Please contact the corresponding author directly for reuse.
