# Supplementary material for: Novel cellular functions of Cys2-His2 zinc finger proteins in anthracnose development and dissemination on pepper fruits by Colletotrichum scovillei
Source: mBio. 2024 Sep 9;15(10):e00667-24. doi: 10.1128/mbio.00667-24 (PMC11481868; doi:10.1128/mbio.00667-24)
Supplement: Supplemental figures — Fig. S1 to S9. [file mbio.00667-24-s0001.pdf]

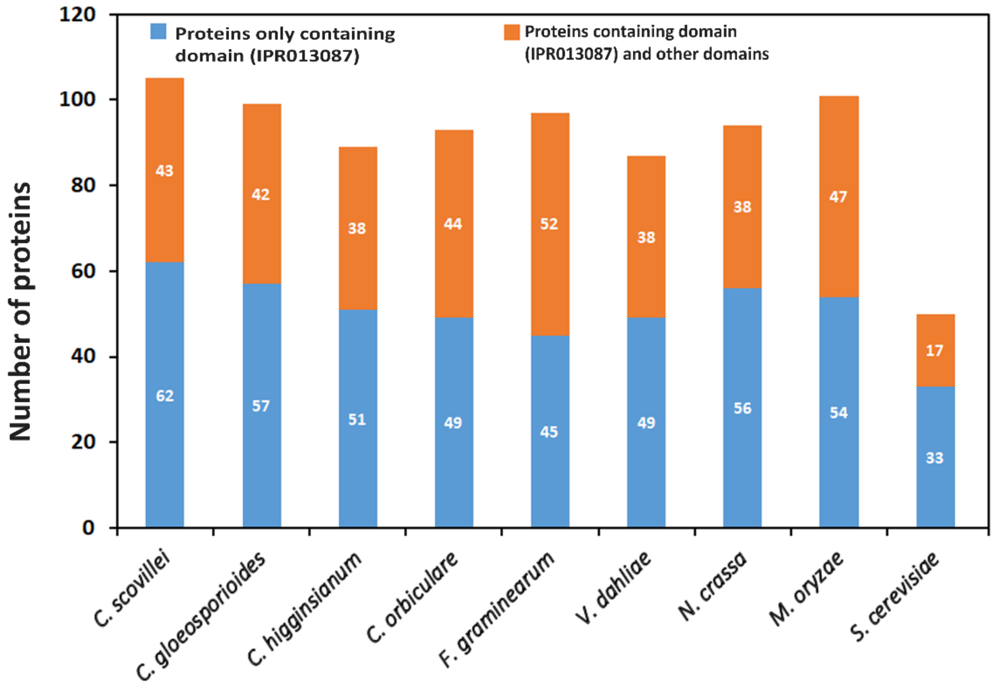

Fig. S1 Numbers of C_2H_2 zinc finger proteins in fungi. The C_2H_2 zinc finger proteins were isolated by searching the C_2H_2 zinc finger domain (InterPro, IPR013087). Blue and orange color represents the number of proteins containing only C_2H_2 zinc finger domain and C_2H_2 zinc finger domain and other domains, respectively.

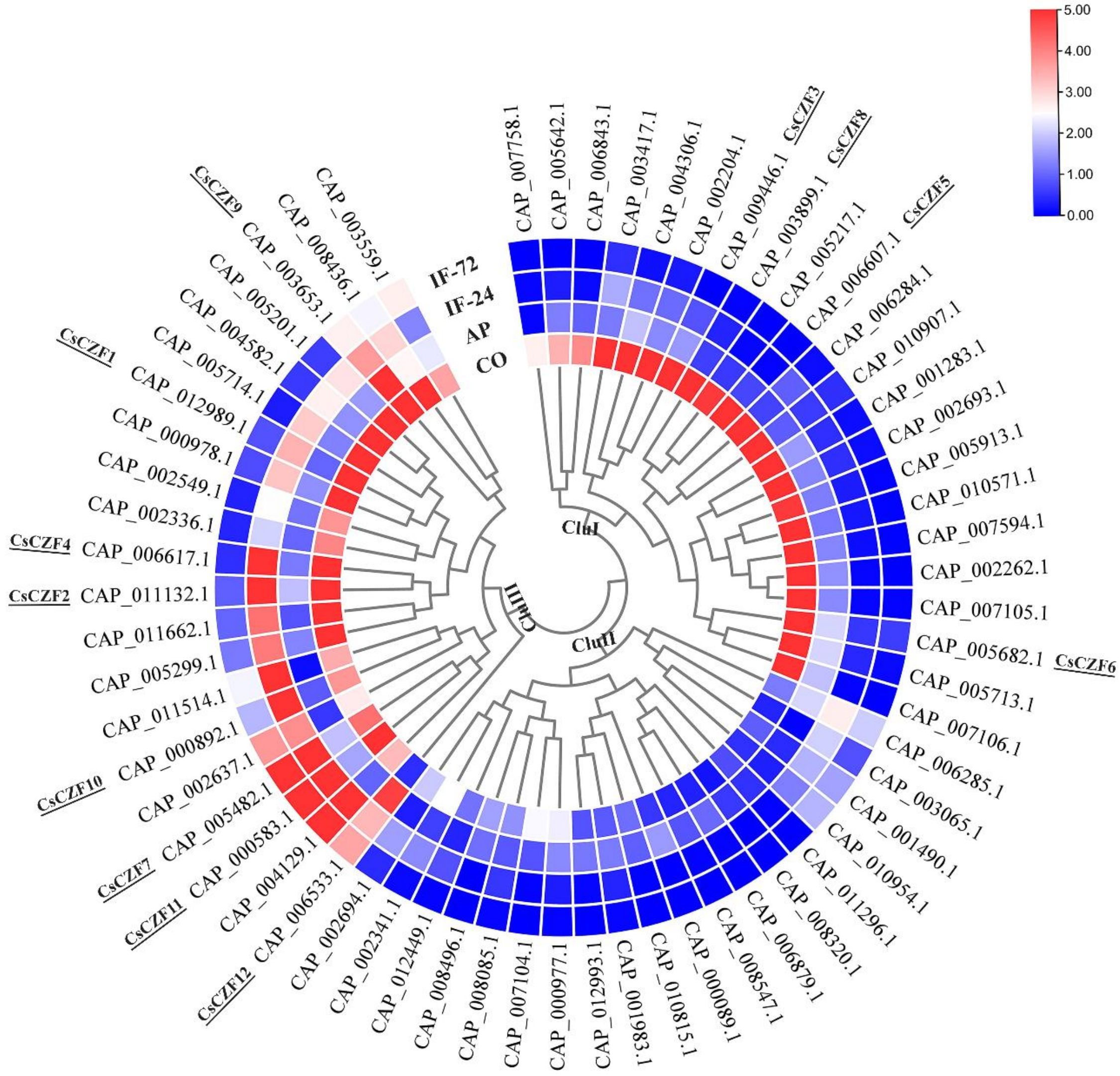

Fig. S2 Expression profiles of the 62 C_2H_2 zinc finger genes using qRT-PCR. The 62 C_2H_2 zinc finger genes were categorized into three clusters based on their expressions in mycelia (MY), conidia (CO, that do not contain mycelia), appressoria (AP), infected plant tissue at 24 h (IF-24) and 72 h (IF-72). The gene expressions in CO, AP, IF-24, and IF-72 were referenced to that in MY. The black dots beside gene names indicate the genes selected for functional characterization.

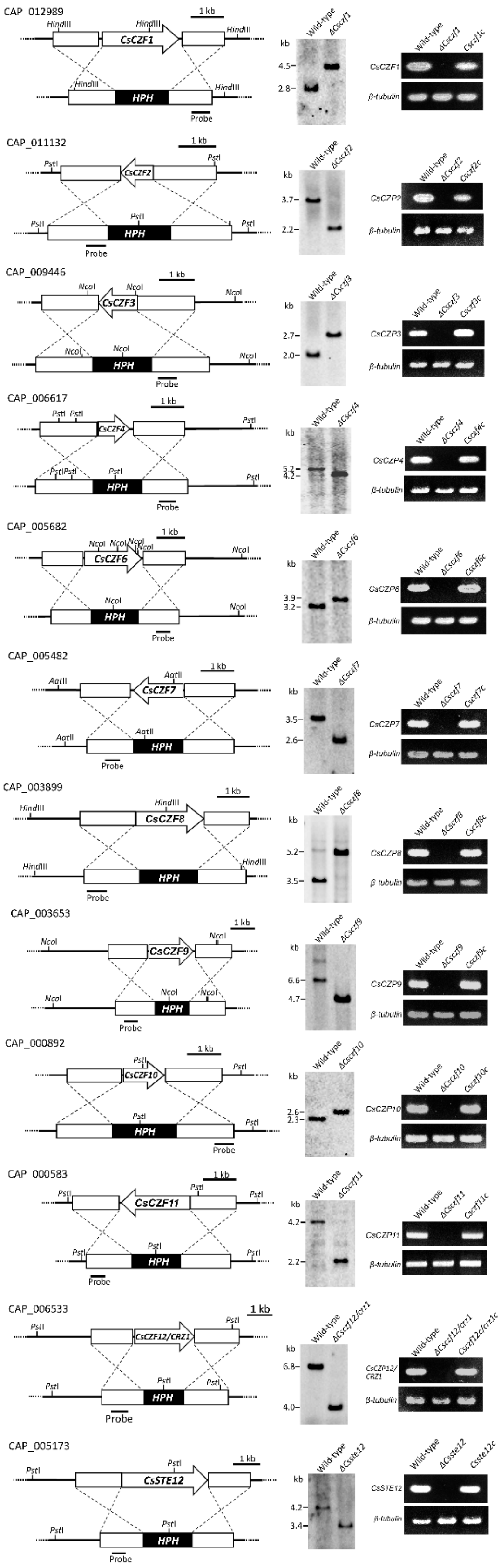
CAP_012989
CsCZF1
HPH
Probe
1 kb
4.5
2.8
CAP_011132
CsCZF2
3.7
2.2
CAP_009446
CsCZF3
2.7
2.0
CAP_006617
CsCZF4
5.2
4.2
CAP_005682
CsCZF6
3.9
3.2
CAP_005482
CsCZF7
3.5
2.6
CAP_003899
CsCZF8
5.2
3.5
CAP_003653
CsCZF9
6.6
4.7
CAP_000892
CsCZF10
2.6
2.3
CAP_000583
CsCZF11
4.2
2.2
CAP_006533
CsCZF12/CRZ1
6.8
4.0
CAP_005173
CsSTE12
4.2
3.4
Wild-type
β-tubulin

Fig. S3 Generation of targeted gene deletion mutants. The genes were deleted via the homology-dependent replacement (left panel). The deletion mutants were confirmed by Southern blotting (middle panel). The complemented strains for each gene were confirmed by using RT-PCR (right panel). The *C. scovillei β-tubulin* gene was expressed as a reference. Targeted deletion mutant of *CsCZF5* (*CAP_006607.1*) was previously generated (1).

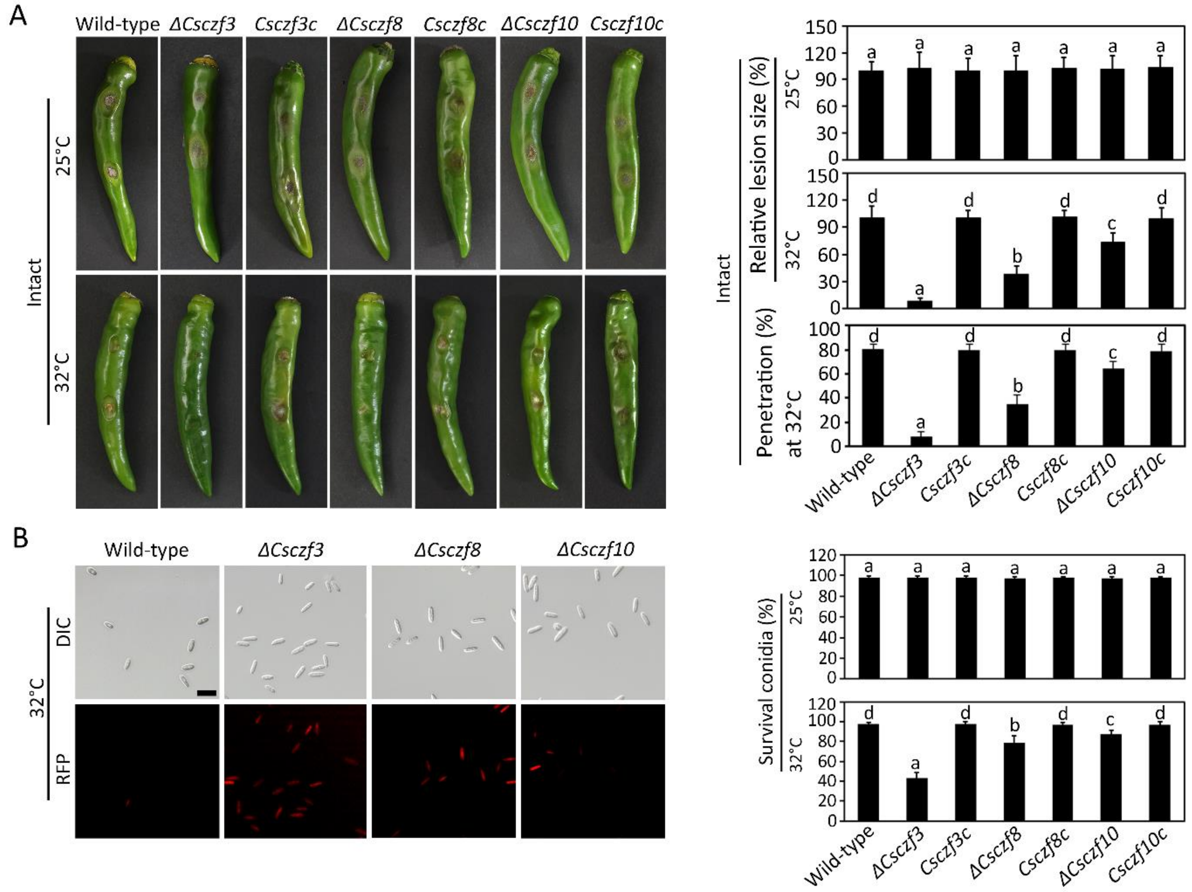

Fig. S4 Roles of *CsCZFs* in conidium viability and pathogenicity under heat shock. (A) Pathogenicity assay on intact pepper fruits under heat shock. Conidial suspensions (5×10^5 mL^{-1}) were inoculated to intact pepper fruits and incubated in a humid box at 25 and 32°C for 8 days. Lesion size was measured by using the Image J. In the penetration assay, conidial suspensions (5×10^4 mL^{-1}) were inoculated to intact pepper fruits and incubated in a humid box at 32°C for 2 days. Penetration rate was evaluated in at least 100 conidia per replicate and performed in 3 independent experiment with 3 replicates per experiment. (B) Assessment of conidium viability. Conidia harvested from 7-day old OMA were stained with phloxine B staining. At least 100 conidia were examined per replicate. Scale bar = 10 μm. Significant difference was estimated by Duncan's test ($P < 0.05$) and indicated by different lowercase letters.

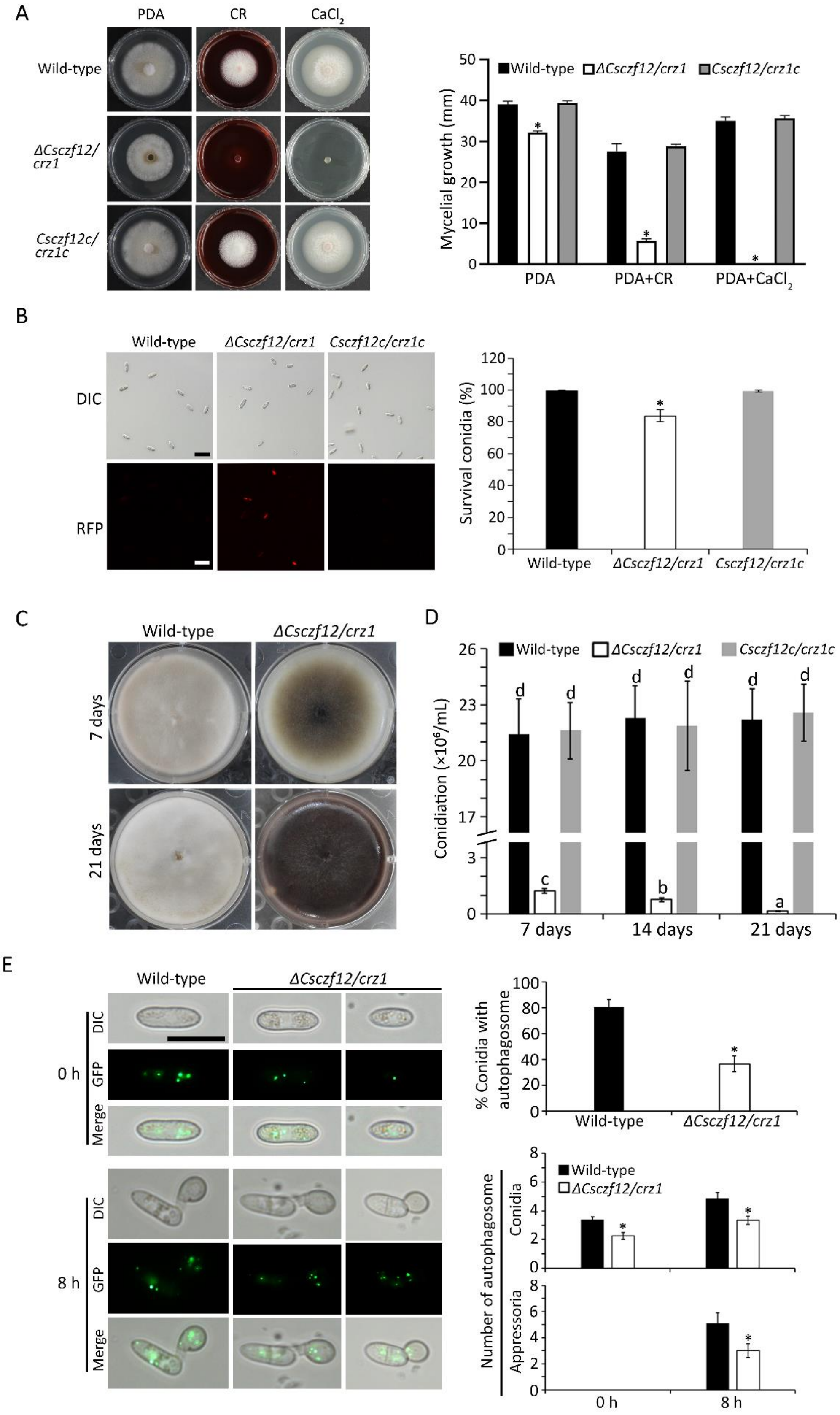
A
PDA
CR
CaCl2
Wild-type
ΔCsczf12/crz1
Csczf12c/crz1c
Wild-type
ΔCsczf12/crz1
Csczf12/crz1c
Mycelial growth (mm)
50
40
30
20
10
0
PDA
PDA+CR
PDA+CaCl2
B
Wild-type
ΔCsczf12/crz1
Csczf12c/crz1c
DIC
RFP
Survival conidia (%)
120
100
80
60
40
20
0
Wild-type
ΔCsczf12/crz1
Csczf12/crz1c
C
Wild-type
ΔCsczf12/crz1
7 days
21 days
D
Wild-type
ΔCsczf12/crz1
Csczf12c/crz1c
Conidiation (×10^6/mL)
26
23
20
17
3
0
d
c
b
a
7 days
14 days
21 days
E
Wild-type
ΔCsczf12/crz1
DIC
GFP
Merge
0 h
8 h
% Conidia with autophagosome
100
80
60
40
20
0
Wild-type
ΔCsczf12/crz1
Number of autophagosome
Conidia
Appressoria
8
6
4
2
0
Wild-type
ΔCsczf12/crz1
0 h
8 h

Fig. S5 Roles of *CsCZF12/CRZ1* in viability, autophagy, and stress response. (A) Mycelial growth on cell wall integrity and calcium stresses. Hyphal agar plugs (5 mm in diameter) obtained from 3-day old MMA were inoculated to PDA containing 300 ppm Congo red and 0.2 M $CaCl_2$ and incubated at 25°C and darkness for 5 days. Mycelial growth was assessed by measuring the colony diameter. (B) Assessment of conidium viability. Conidia harvested from 7-day old OMA were stained with phloxine B staining. At least 100 conidia were examined per replicate. Scale bar = 20 µm. (C) Visualization of mycelial autolysis. Hyphal agar plugs (5 mm in diameter) obtained from 3-day old MMA were inoculated to OMA and cultured at 25°C and lightness for 7 and 21 days. (D) Conidiation assay. Hyphal agar plugs (5 mm in diameter) obtained from 3-day old MMA were inoculated to OMA and cultured at 25°C and lightness. Conidiation was evaluated by counting conidia from OMA harvested with 5 mL sterilized distilled water. (E) Visualization of GFP:CsATG8 in conidium and appressorium. Scale bar = 10 µm.

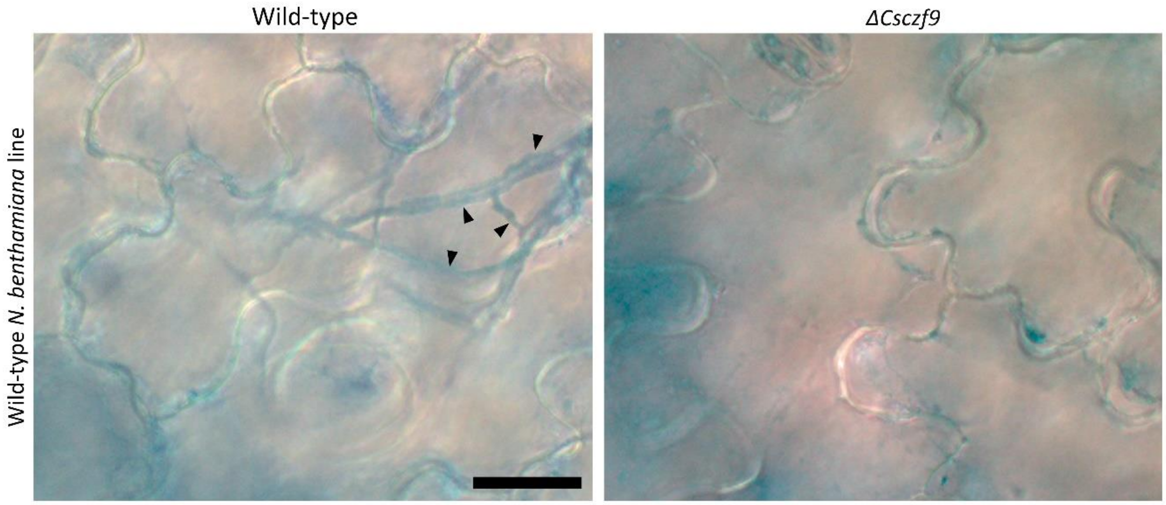

Fig. S6 Visualization of invasive hyphae. Hyphal agar plugs (2 mm in length) from 4-day water agar medium were inoculated to leaves with artificial wound of 8-week-old wild-type *N. benthamiana* line. After 3 days, infected leaves were fixed in a solution containing acetic acid, chloroform, and methanol and then rehydrated in 70% ethanol (2). The samples were then stained with lactophenol cotton blue staining. Invasive hyphae were indicated with black triangles. Scale bar = 30 µm.

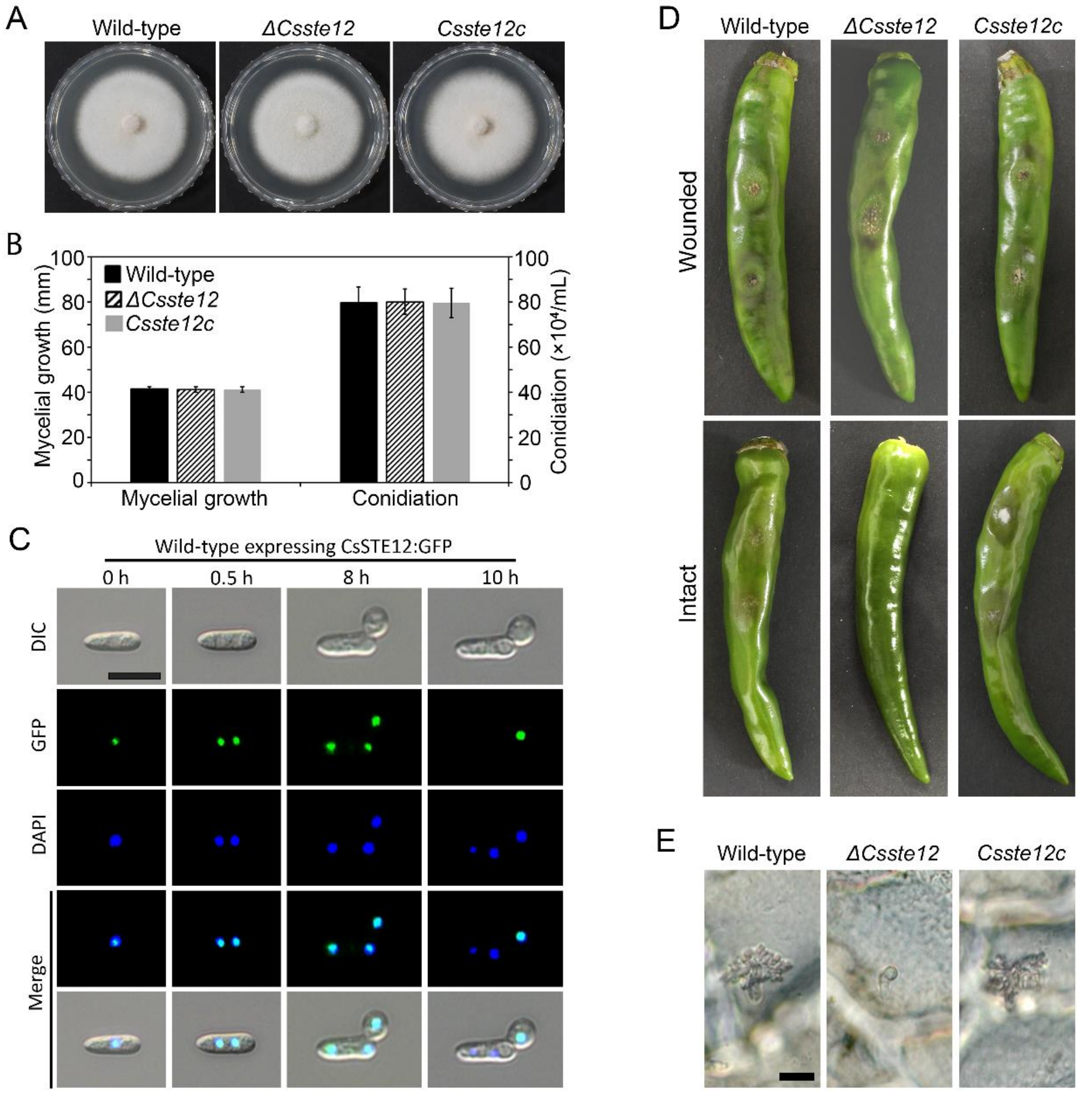

Fig. S7 Functional characterizations of *CsSTE12*. (A) Vsualization of mycelial growth. Mycelial agar plugs (5 mm in diameter) from 3-day old MMA were inoculated to PDA and incubated at 25°C for 4 days. (B) Assessments of mycelial growth and conidiation. Mycelial growth was determined by measuring diameters of colony growth shown in (A). Conidiation was determined by counting conidia harvested with 5 mL distilled water form 7-day-old V8. (C) Subcellular localization of CsSTE12:GFP. Conidial suspension of wild-type strain expressing CsSTE12:GFP obtained from 7-day-old OMA were dropped onto the hydrophobic surface of coverslips and

incubated in a humid box at 25°C. Nucleus was stained using DAPI. Scale bar = 10 μm. (D) Pathogenicity assays. Conidial suspensions were inoculated to wounded and intact pepper fruits and incubated in humid boxes at 25°C for 6 and 9 days, respectively. (E) Penetration assay. Conidial suspensions were inoculated to intact pepper fruits and incubated in humid boxes at 25°C for 2 days. CO, AP, and DS indicates conidium, appressorium, and dendroid structure, respectively. (C and E) Scale bar = 10 μm.

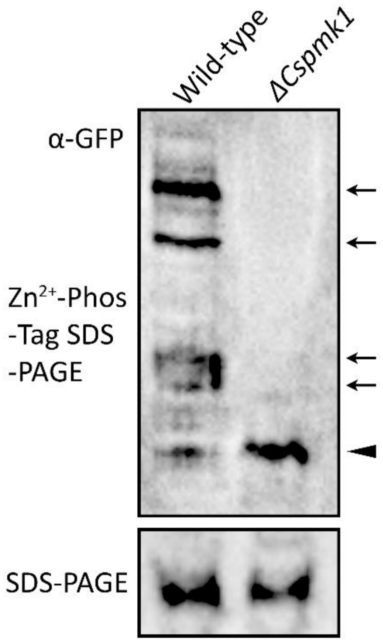

Fig. S8 Phosphorylation of CsCZF9 by CsPMK1. Conidia of the wild-type and *ΔCspmk1* strains expressing CsCZF9:GFP were harvested from 7-day-old OMA and used for total protein extraction. Total protein was separated in SDS-PAGE and Zn^{2+}-Phos-Tag SDS-PAGE and transferred to PVDF membranes, which were further detected with anti-GFP antibody. Black arrow and triangle indicate phosphorylated and non-phosphorylated form of CsCZF9 by CsPMK1, respectively.

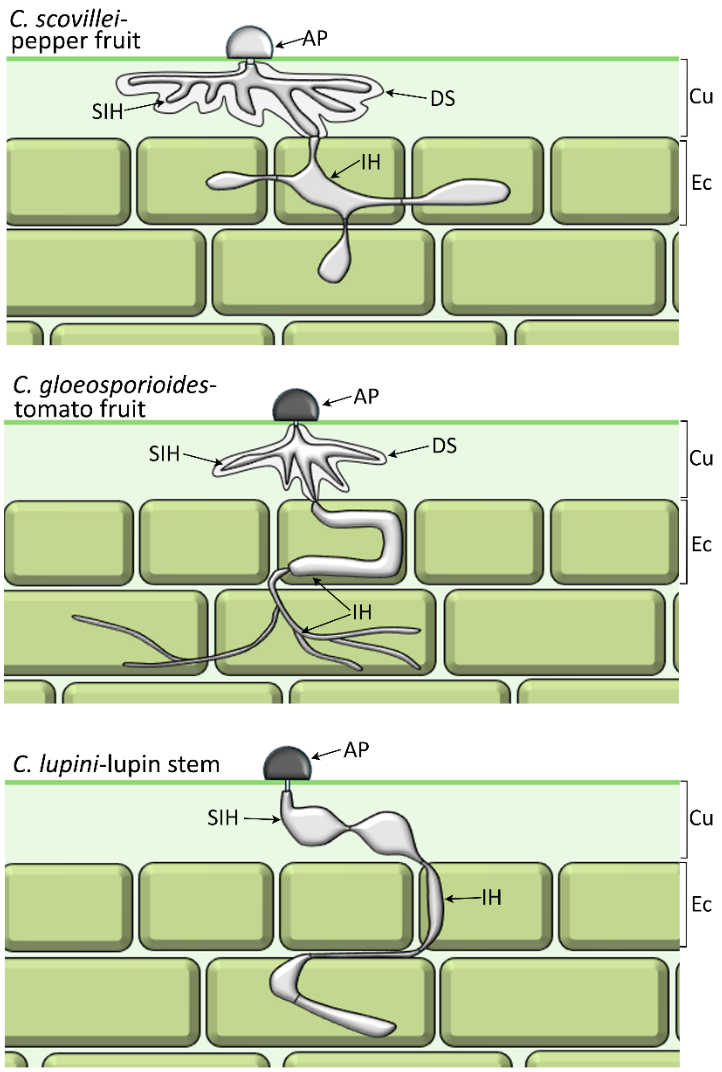

Fig. S9 Infections of *C. scovillei*, *C. gloeosporioides*, and *C. lupini* in pepper fruit, tomato fruit, and lupin stem, respectively (3-5). Before invading to host epidermal cells, both *C. scovillei* and *C. gloeosporioides* forms highly branched hyphae, visualized as dendroid structures (DSs), in the subcuticular layer of fruit. The DS of *C. gloeosporioides* become quiescent in immature tomato fruit. Distinctly, *C. lupini* develops unbranched and swollen hyphae in subcuticular layer of lupin stem. AP, SIH, IH, Cu, and Ec indicates appressorium, subcuticular intramural hyphae, invasive hyphae, cuticle, and epidermal cell, respectively.

References

1. Shin J-H, Han J-H, Park H-H, Fu T, Kim KS. 2019. Optimization of polyethylene glycol-mediated transformation of the pepper anthracnose pathogen *Colletotrichum scovillei* to develop an applied genomics approach. Plant Pathol J 35:575–584. doi.org/10.5423/PPJ.OA.06.2019.0171
2. Fu T, Han J-H, Shin J-H, Song H, Ko J, Lee Y-H, Kim K-T, Kim KS. 2021. Homeobox transcription factors are required for fungal development and the suppression of host defense mechanisms in the *Colletotrichum scovillei*-pepper pathosystem. mBio 12:e01620-21. doi.org/10.1128/mbio.01620-21
3. Fu T, Park H-H, Kim KS. 2022. Role of the cAMP signaling pathway in the dissemination and development on pepper fruit anthracnose disease caused by *Colletotrichum scovillei*. Front cell infect microbiol 12:1003195. doi.org/10.3389/fcimb.2022.1003195
4. Alkan N, Friedlander G, Ment D, Prusky D, Fluhr R. 2015. Simultaneous transcriptome analysis of *Colletotrichum gloeosporioides* and tomato fruit pathosystem reveals novel fungal pathogenicity and fruit defense strategies. New Phytol 205:801-815. doi.org/10.1111/nph.13087
5. Guilengue N, Silva MdC, Talhinhas P, Neves-Martins J, Loureiro A. 2022. Subcuticular–intracellular hemibiotrophy of *Colletotrichum lupini* in *Lupinus mutabilis*. Plants 11:3028. doi.org/10.3390/plants11223028
